# Supplementary material for: Metabolic reprogramming of the infant gut by bifidobacteria-based probiotics drives exclusion of antibiotic-resistant pathobionts
Source: Cell Rep Med. 2026 Apr 20;7(5):102752. doi: 10.1016/j.xcrm.2026.102752 (PMC13198307; doi:10.1016/j.xcrm.2026.102752)
Supplement: Document S1. Figures S1–S10 and Tables S1 and S10 [file mmc1.pdf]

Cell Reports Medicine, Volume *7*

Supplemental information

Metabolic reprogramming of the infant gut by bifidobacteria-based probiotics drives exclusion of antibiotic-resistant pathobionts

Ahmed Bargheet, Gaute Hovde Bø, Marit Andrea Klokkhammer Hetland, Museveni Justine, Sabrina John Moyo, Iren Høyland Löhr, Bjørn Blomberg, Nina Langeland, Claus Klingenberg, and Veronika Kuchařová Pettersen

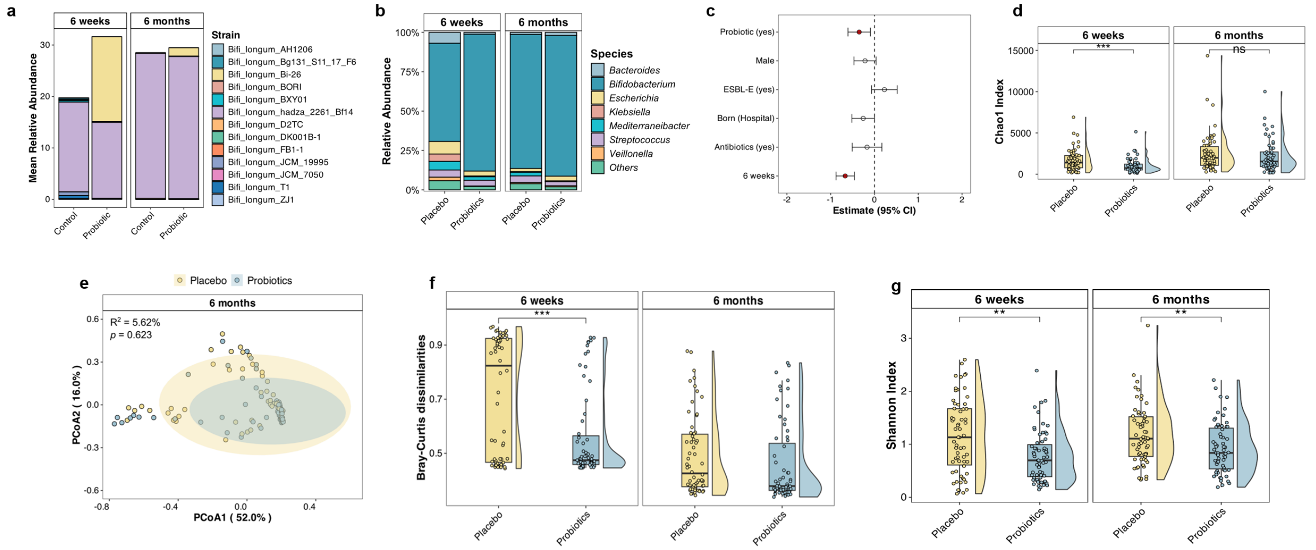

Figure S1. **Analyses of *Bifidobacterium longum* sub-species diversity and microbiota α- and β-diversity.** Related to Figure 1 (a) Relative abundances of *B. longum* strains identified by StrainGE at the 6-week and 6-month time points. (b) Relative abundance of bacterial genera in the placebo and probiotic groups. (c) Impact of selected variables on infant gut microbiota richness, determined by a negative binomial model. (d) Alpha diversity by Chao1 index (Mann–Whitney U test). (e) Principal Coordinate Analysis (PCoA) of Bray–Curtis dissimilarities at 6 months; each point represents one sample. (f) Bray–Curtis dissimilarity to all other samples within the same group at 6 weeks and 6 months; each point represents a sample's mean Bray–Curtis dissimilarity to all other samples in its group (Mann–Whitney U test). (g) Shannon index at 6 weeks and 6 months (Mann–Whitney U test). For Shannon index, the Wilcoxon effect size for 6 weeks = 0.276, magnitude small; for 6 months, effect size = 0.233, magnitude small. Statistically significant results are based on $p < 0.05$. *** $p < 0.001$, ** $p < 0.01$.

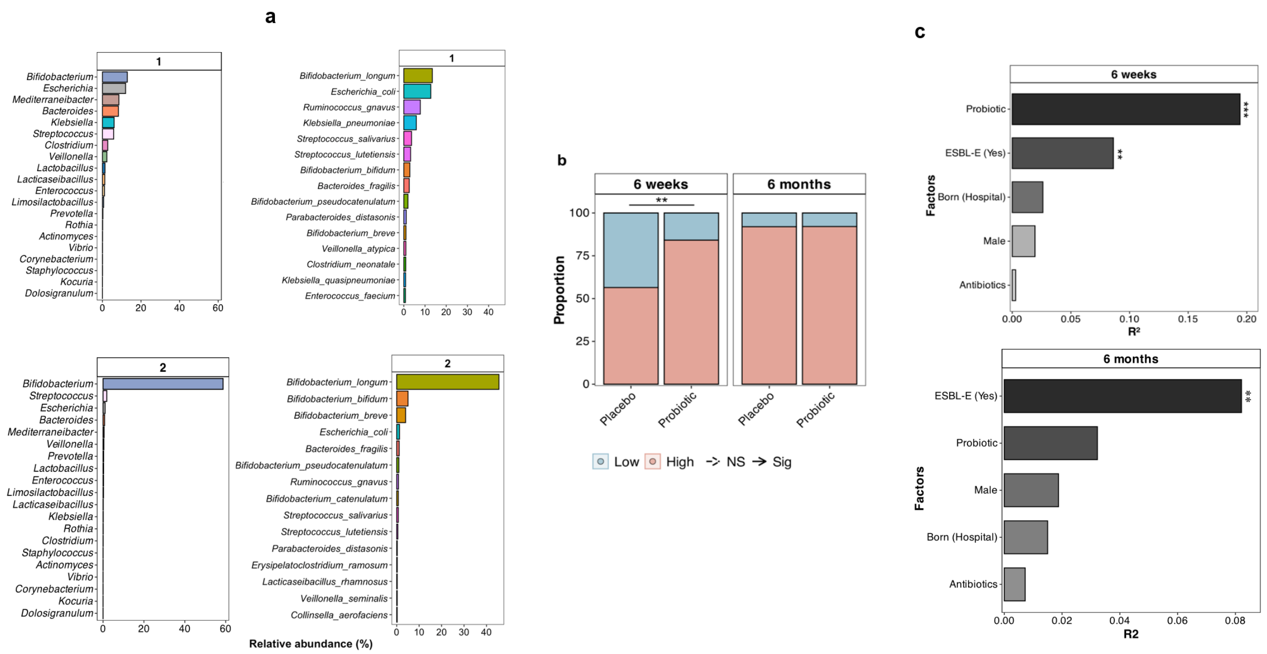

Figure S2. **Unsupervised clustering and Envfit analyses.** Related to Figure 1. a) Microbiota cluster composition identified using k-means clustering, with Cluster 1 ("Low" top row) and Cluster 2 ("High" bottom row) at the genus (left) and species (right) level. (b) Distribution of clusters across groups, with the *p*-value calculated using Fisher's exact test. (c) Factors associated with microbiota community structure, identified using Envfit analysis, at 6 weeks and 6 months of age. Statistical significance: $p < 0.05$. Symbols: $***$ $p < 0.001$; $**$ $p < 0.01$; $*$ $p < 0.05$.

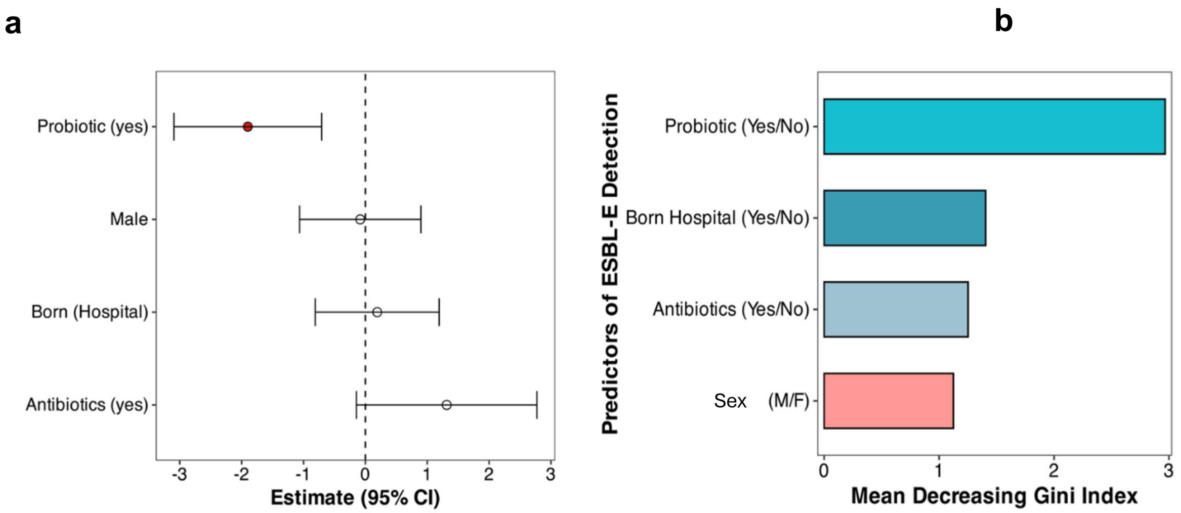

Figure S3. **Impact of probiotics on ESBL-E carriage.** Related to Figure 2. The impact of selected variables on the ESBL-E carriage as determined by generalized linear modelling (a) and random forest (b). The random forest analysis does not indicate which specific binary outcome a variable is associated with, but it identifies which variables are most important for prediction. Statistical significance: $p < 0.05$

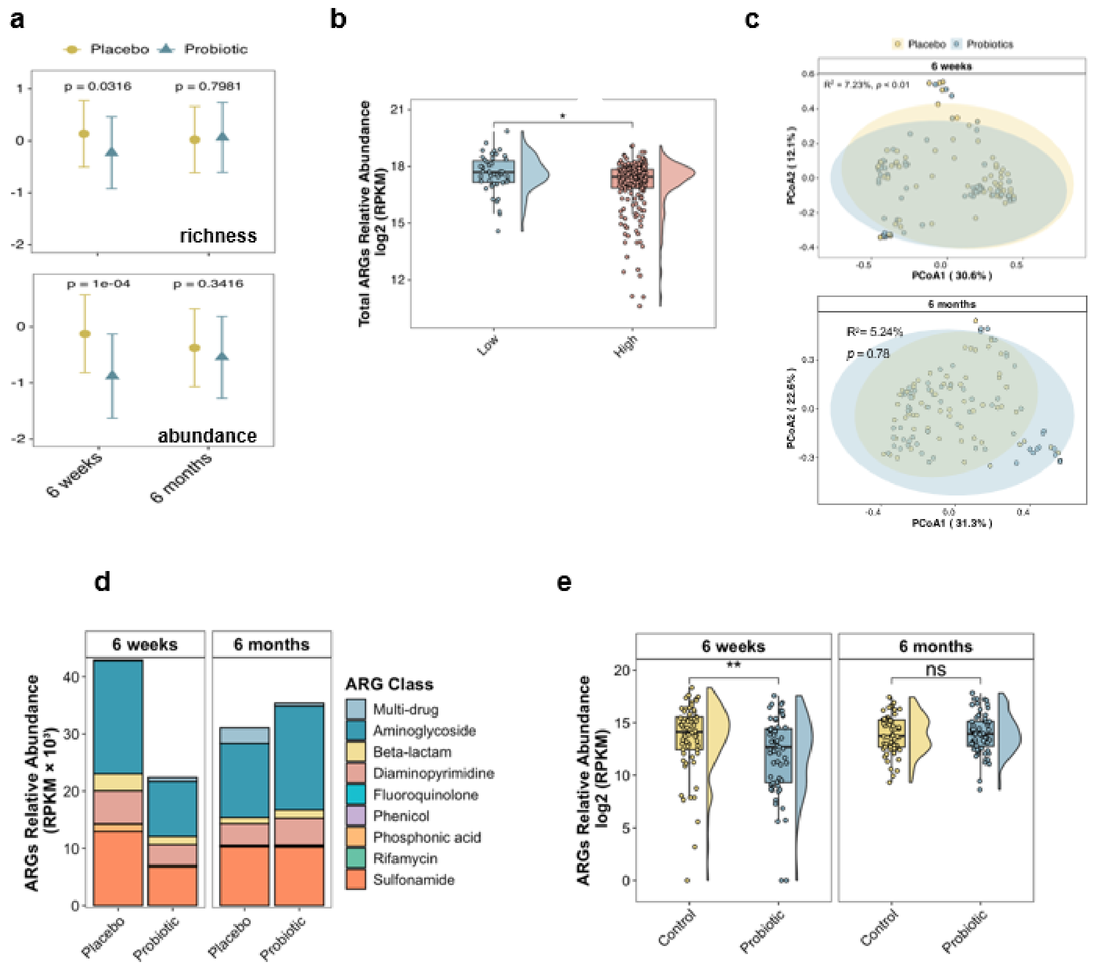

Figure S4. The influence of probiotics on the resistome. Related to Figure 2. a) Estimated marginal means of probiotic impact on resistome richness and relative abundance across time points. b) Comparison of antibiotic resistance genes (ARGs) relative abundance between microbiota clusters. Reads per kilobase per million mapped reads (RPKM), with each point representing a sample. p-values were computed using the Mann-Whitney U test. c) Principal Coordinate Analysis (PCoA) illustrating resistome beta diversity differences between the probiotic and placebo groups at 6 weeks and 6 months, visualized through Jaccard dissimilarity comparisons. Panels (d) and (e) reproduce the analyses shown in Figure 2 of the main text but restricted to the 33 clinically relevant ARGs detected in the 32 isolate-metagenome-matched samples. This subset highlights resistome changes attributable specifically to ARGs carried by ESBL-producing isolates recovered from the same individuals. d) Relative abundance of ARGs in RPKM, stratified by ARG classes. The multidrug-resistant class refers to ARGs annotated as conferring resistance to two or more antibiotics. (e) Comparison of ARG abundance between groups, with each point representing a sample. Statistical significance: $p < 0.05$ (∗), ∗∗ $p < 0.01$

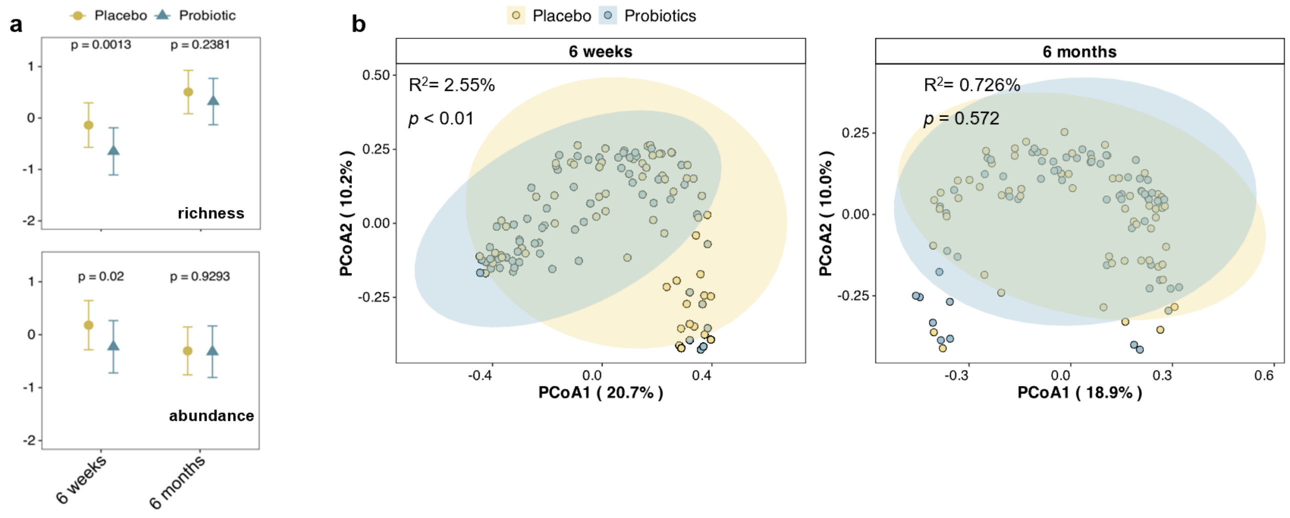

Figure S5. The influence of probiotics on the mobilome. Related to Figure 2. a) Estimated marginal means of probiotic impact on mobilome richness and relative abundance across time points. b) Principal Coordinate Analysis (PCoA) illustrating mobilome beta diversity differences between the probiotic and placebo groups at 6 weeks and 6 months, visualized through Jaccard dissimilarity comparisons. Statistical significance: $p < 0.05$

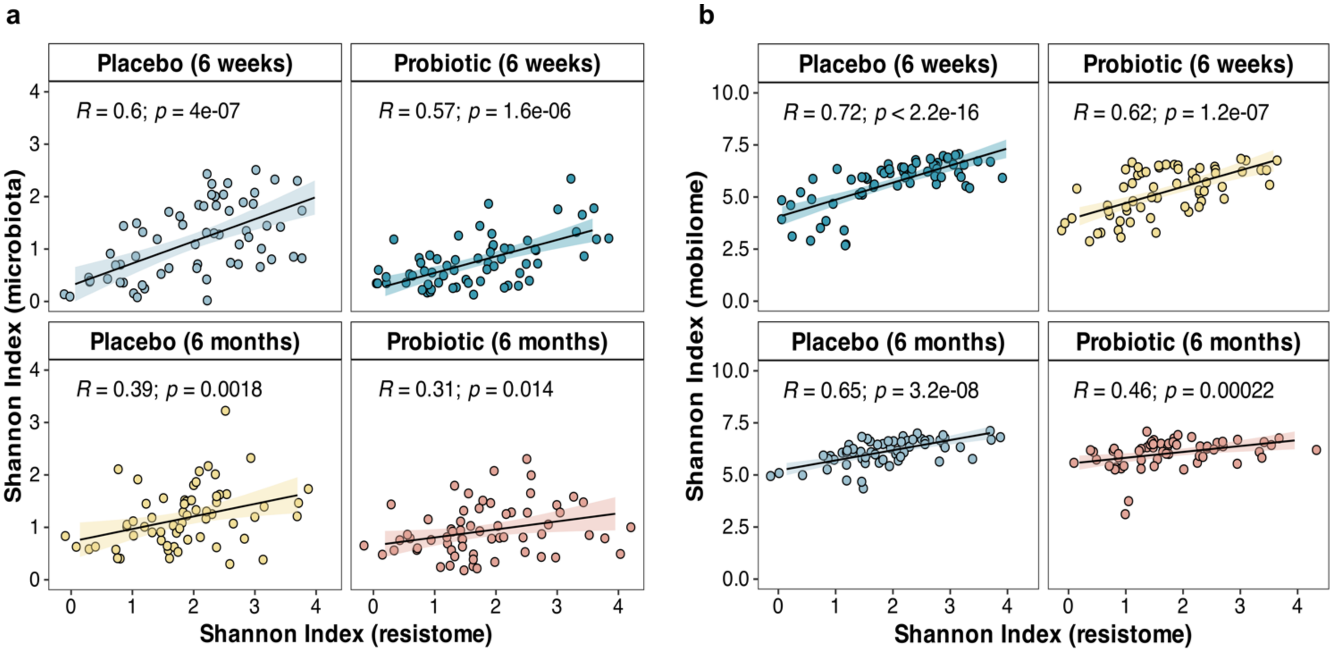

Figure S6. Correlation analysis. Related to Figure **2.** Spearman correlation analysis between resistome diversity and microbiota diversity (a) and between resistome diversity and mobilome diversity (b). The strength of correlation estimated by R values: 0.10-0.30 weak, 0.30-0.50 moderate, 0.5-0.7 strong, 0.7-1 very strong.

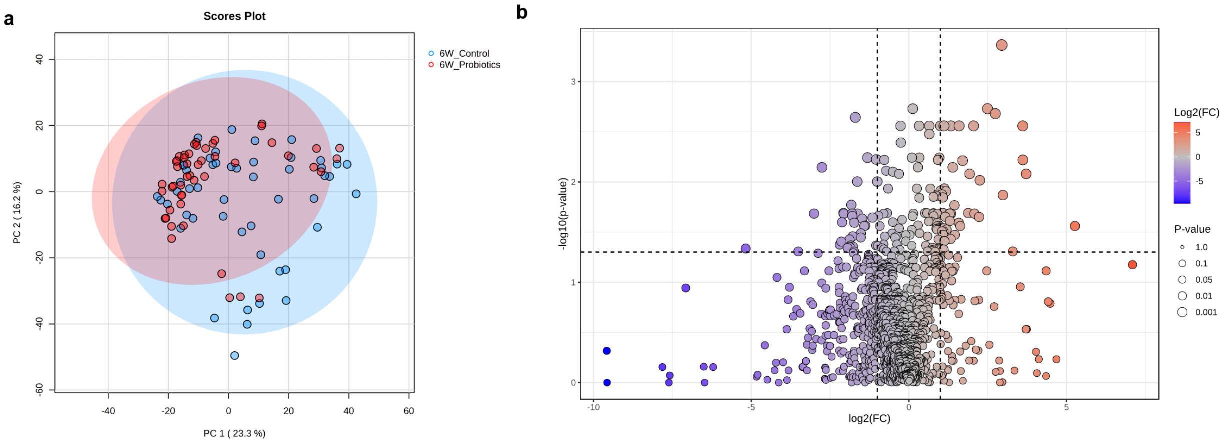

Figure S7. Comparison of the fecal metabolome of placebo and probiotic groups at 6 weeks. Related to Figure 3. a) Principal component analysis (PCA) of 1188 detected metabolic features ($R^2 = 0.07218$, $p = 0.004$) using Euclidean distance matrix. b) Volcano plot describing differences in metabolic features between probiotic and placebo groups. In the probiotic group, 45 features showed significantly increased abundance, whereas 37 showed significantly decreased abundance. Settings used in MetaboAnalyst: *p*-value set to 0.05 (Mann-Whitney U test), accounting for unequal group variance, applying false discovery rate correction, and a fold change cutoff of 2.0. The untargeted metabolomic data were normalized by median, $\log_{10}$ transformed, and Pareto scaled.

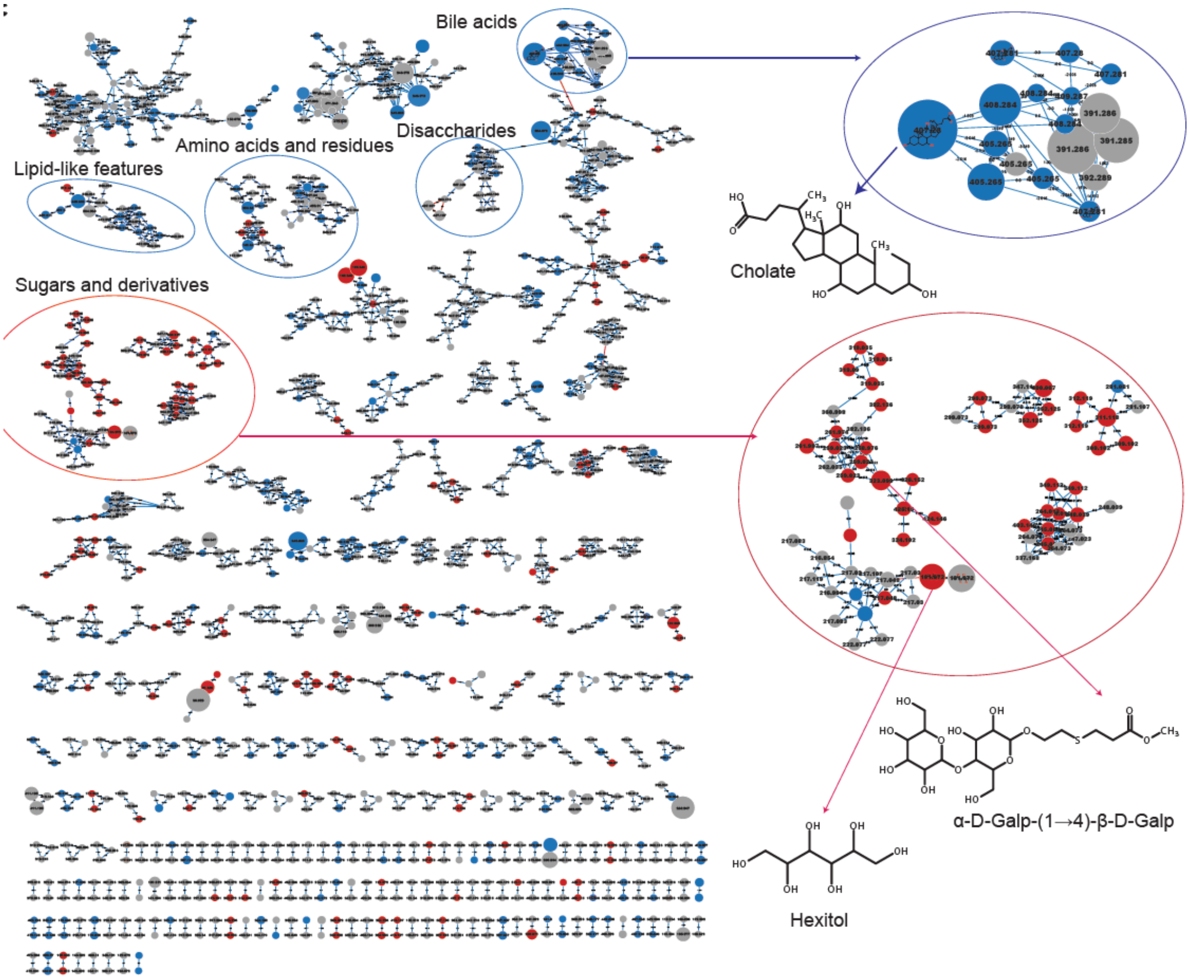

Figure S8 Feature-based molecular networking of untargeted metabolomic data derived from 6 weeks ProRIDE stool samples. Related to Figure 3**.** Clustering was based on cosine and MS1 similarities between features, indicating related fragments and precursor ions, respectively. Node size reflects the sum of the precursor area for each feature. Significantly elevated features in the high Bifidobacterium cluster group are labelled red, while significantly decreased features are labelled blue (Mann-Whitney U test, unequal group variance, false discovery rate, and fold change cutoff 2.0, after median normalization, log10 transformation, and Pareto scaling).

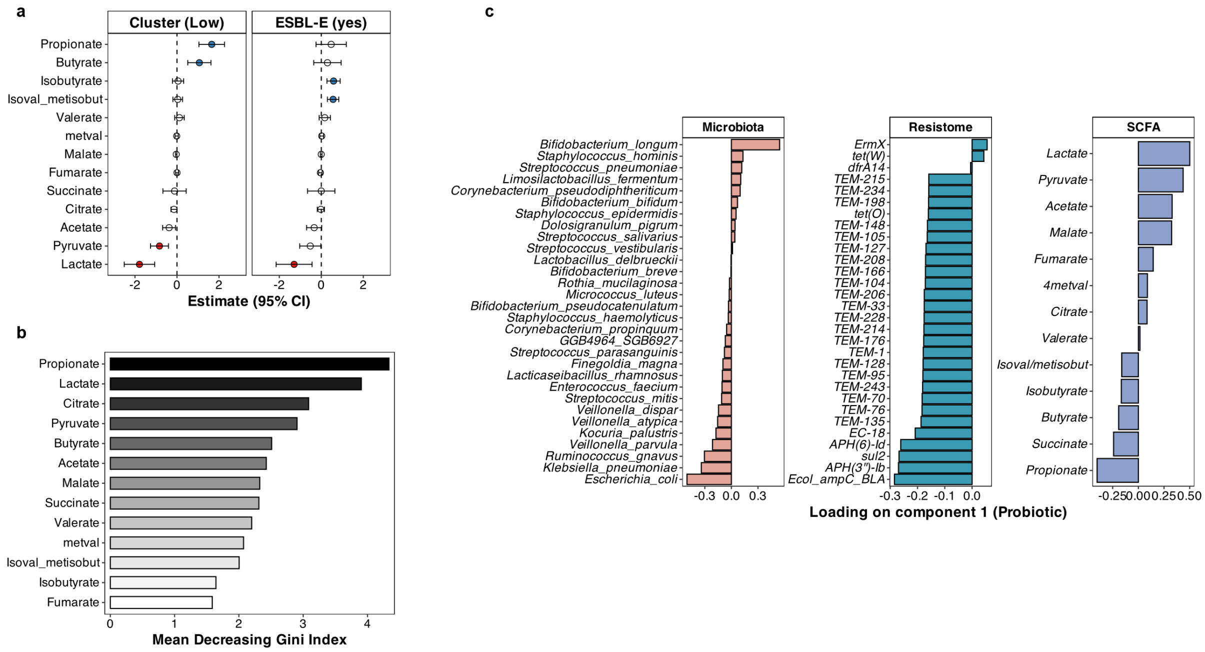

Figure S9 Targeted metabolomics data analysis. Related to Figure 4. **(a)** Differential abundance of the metabolites identified using linear model (Benjamini-Hochberg adjusted p values). (**b**) The importance of SCFAs on microbiota composition as determined by random forest. (**c**) SCFA loadings from DIABLO analysis for the probiotic component.

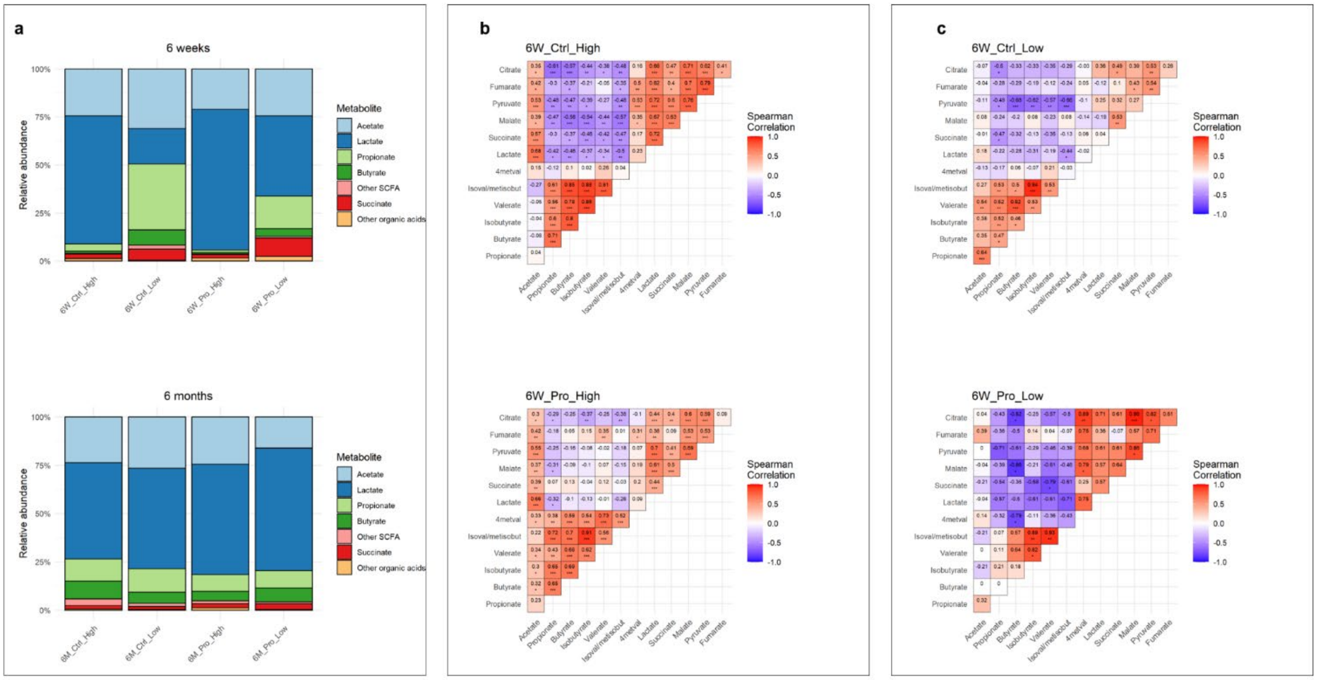

Figure S10. Dynamics of SCFAs and OAs based on *Bifidobacterium* spp. abundance. Related to Figure **4.** a) Relative proportion of SCFAs and organic acids in samples from 6 weeks and 6 months for the probiotic and control group, and with either high or low *Bifidobacterium* spp. abundance. Correlation heatmaps showing Spearman correlations between metabolites for b) "High" and c) "Low" groups at 6 weeks. Asterisks indicate significance levels (*p < 0.05, **p < 0.01, ***p < 0.001).

Table S1. Baseline characteristics of participants. Related to STAR Methods.

Variables	Placebo (N=72)	Probiotics (N=80)
**Mother and delivery**		
Maternal age, median (IQR), years	25 (22.0 - 29.3)	26.5 (22.0 – 31.0)
Maternal HIV infection	2.8 % (2/72)	5.0 % (4/80)
Mode of delivery – n (%)		
• Vaginal	95.8 % (69/72)	97.5 % (78/80)
• Caesarean section	4.2 % (3/72)	2.5 % (2/80)
Birthplace – n (%)		
• Haydom Lutheran Hospital	43.1 % (31/72)	40.0 % (32/80)
• Other health facility	22.2 % (16/72)	25.0 % (20/80)
• At home	34.7% (25/72)	35.0 % (28/80)
**Child**		
Female sex – n (%)	50.0 % (36/72)	48.8 % (39/80)
Birth weight, median (IQR) - kg	3.3 (3.0 – 3.5)	3.3 (3.0 – 3.5)
Birth length, median (IQR) - cm	48.5 (47.0 – 49.0)	48.0 (47.0 – 49.0)
Apgar score at 5 min, median (IQR)*	10 (10-10)	10 (10-10)
Apgar score at 5 min < 9 – n (%)*	0 (0%)	0 (0%)
Apgar score at 5 min		
- 9	3 (4.2 %)	2 (2.5 %)
- 10	27 (37.5 %)	29 (36.3 %)
- Not recorded	42 (58.3 %)	49 (61.3 %)
**Breastfeeding**		
At 6 weeks – no. (%) †	100.0 % (72/72)	100.0 % (80/80)
At 6 months – no. (%) †	94.4 % (68/72)	95.0 % (76/80)
**Caregiver-reported adherence during the 4-week intervention**		
Study drug given daily – no. (%)	84.7 % (61/72)	95.0 % (76/80)
Forgot a dose 1-6 days – no. (%)	13.9 % (10/72)	3.8% (3/80)
Forgot a dose 7-21 days – no. (%)	0.0 % (0/72)	1.3% (1/80)
Forgot a dose ≥ 22 days– no. (%)	1.4% (1/72)	0.0 % (0/80)
**ESBL-E gut colonisation**		
6 weeks	25.0% (18/72)	5.0% (4/80)
6 months	25.0% (18/72)	15.0% (12/80)

Table S1. Baseline characteristics of participants - continuation

Variables	Placebo (N=72)	Probiotics (N=80)
**Death or hospitalisation to 6 m**		
Death* (n, %)	0 (0.0%)	0 (0.0%)
Hospitalisation to 6 m	4 (5.6%)	1 (1.3%)
Δ weight Z birth→6 m, mean (SD)	−0.37 ± 8.10	−0.64 ± 7.60
Δ length Z birth→6 m, mean (SD)	−0.43 ± 1.29	−0.25 ± 1.19
**Caregiver-reported adverse events**		
Gastrointestinal adverse events**	7 (9.7%)	6 (7.5%)
Reported visit to an outpatient clinic (OPC) up to 6 m	23 (31.9%)	22 (27.5%)
OPC ≤6 weeks	8 (11.1%)	8 (10.0%)
OPC 6w–6m	18 (25.0%)	15 (18.8%)
Any antibiotic therapy up to 6 m	23 (31.9%)	27 (33.8%)
Hospitalisation with suspected infection	4 (5.6%)	1 (1.3%)

* No death or blood culture–positive hospitalizations were recorded

** Including diarrhea, constipation, abdominal pain, bloating, and bloody stools

Table S10. Bacterial species with pathogenic potential reported by previous studies. Related to Figure 1.

**Pathobiont**	**Source**
Acinetobacter baumannii	Shao.et,al (2019)[1]; Murray et al., 2022[2]
Campylobacter jejuni	Burnham et al, 2018[3]
Clostridium perfringens	Shao.et,al (2019)
Enterobacter cloacae	Shao.et,al (2019)
Enterococcus faecalis	Shao.et,al (2019); Murray et al., 2022
Enterococcus faecium	Shao.et,al (2019)
Escherichia coli	Murray et al., 2022
Haemophilus influenzae	Murray et al., 2022
Klebsiella oxytoca	Shao.et,al (2019)
Klebsiella pneumoniae	Shao.et,al (2019); Murray et al., 2022
Morganella morganii	Murray et al., 2022
Pseudomonas aeruginosa	Shao.et,al (2019); Murray et al., 2022
Salmonella enterica	Murray et al., 2022
Serratia marcescens	Murray et al., 2022
Staphylococcus aureus	Murray et al., 2022
Staphylococcus epidermidis	Shao.et,al (2019)
Streptococcus parasanguinis	Shao.et,al (2019)
Streptococcus pneumoniae	Murray et al., 2022

REFERENCES

1. Shao, Y., *et al.* Stunted microbiota and opportunistic pathogen colonization in caesarean-section birth. *Nature* **574**, 117–121 (2019).
2. Murray, C.J., *et al.* Global burden of bacterial antimicrobial resistance in 2019: a systematic analysis. *The Lancet* **399**, 629–655 (2022).
3. Burnham, P.M. & Hendrixson, D.R. Campylobacter jejuni: collective components promoting a successful enteric lifestyle. *Nature Reviews Microbiology* **16**, 551–565 (2018).
