# Supplementary material for: Metabolic reprogramming of the infant gut by bifidobacteria-based probiotics drives exclusion of antibiotic-resistant pathobionts
Source: Cell Rep Med. 2026 Apr 20;7(5):102752. doi: 10.1016/j.xcrm.2026.102752 (PMC13198307; doi:10.1016/j.xcrm.2026.102752)
Supplement: Document S2. Article plus supplemental information [file mmc16.pdf]

Cell Reports
Medicine

Article

Metabolic reprogramming of the infant gut by bifidobacteria-based probiotics drives exclusion of antibiotic-resistant pathobionts

Graphical abstract

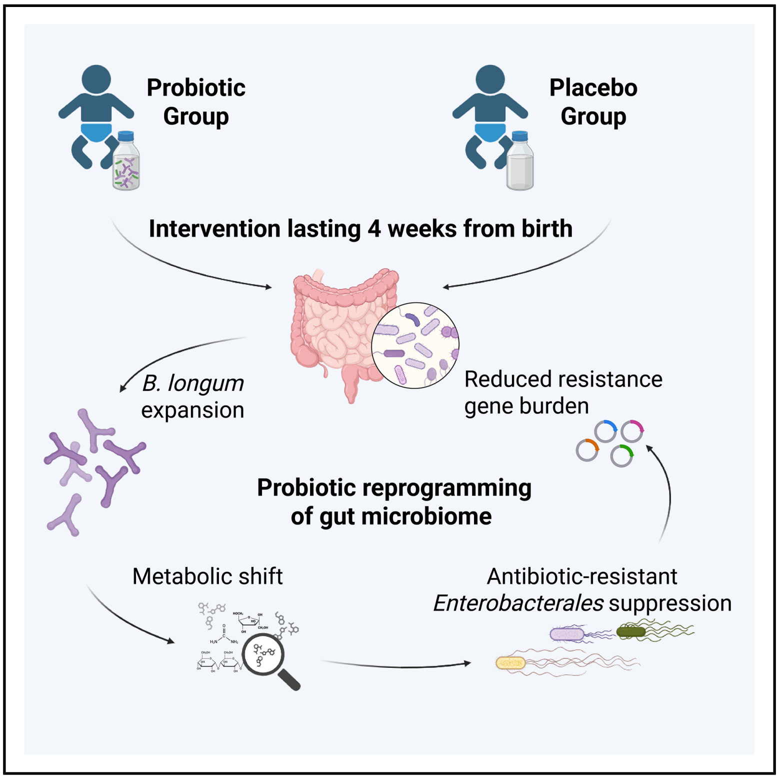

Highlights

- Probiotics increase gut colonization by *Bifidobacterium* spp. in breastfed infants
- *B. longum* expansion suppresses antibiotic-resistant *Enterobacterales*
- Metabolic shifts mediate reduced antibiotic resistance burden

Authors

Ahmed Bargheet, Gaute Hovde Bø, Marit Andrea Klokkhammer Hetland, ..., Nina Langeland, Claus Klingenberg, Veronika Kuchařová Pettersen

Correspondence

veronika.k.pettersen@uit.no

In brief

Bargheet et al. show that probiotic supplementation reshapes the gut microbiome toward *Bifidobacterium* dominance, alters fermentation metabolism, and suppresses antibiotic-resistant *Enterobacterales* in full-term infants in rural Tanzania. Multi-omics analyses reveal a reduced burden of antibiotic-resistance genes and link metabolic reprogramming to lower antibiotic resistance in early life.

Bargheet et al., 2026, Cell Reports Medicine 7, 102752
May 19, 2026 © 2026 The Author(s). Published by Elsevier Inc.
https://doi.org/10.1016/j.xcrm.2026.102752

Cell Reports Medicine

CellPress
OPEN ACCESS

Article

Metabolic reprogramming of the infant gut by bifidobacteria-based probiotics drives exclusion of antibiotic-resistant pathobionts

Ahmed Bargheet,[1,2,3] Gaute Hovde Bø,[1,2,3] Marit Andrea Klokkhammer Hetland,[4,5] Museveni Justine,[2,3,6,7] Sabrina John Moyo,[8,9] Iren Høyland Löhr,[4,5] Bjørn Blomberg,[8,10] Nina Langeland,[8,10,11] Claus Klingenberg,[2,12] and Veronika Kuchařová Pettersen[1,2,3,13,*]

[1]Host-Microbe Interaction Research Group, Department of Medical Biology, Faculty of Health Sciences, UiT The Arctic University of Norway, Tromsø, Norway

[2]Research Group for Child and Adolescent Health, Department of Clinical Medicine, Faculty of Health Sciences, UiT The Arctic University of Norway, Tromsø, Norway

[3]Center for New Antibacterial Strategies, UiT The Arctic University of Norway, Tromsø, Norway

[4]Department of Medical Microbiology, Stavanger University Hospital, Stavanger, Norway

[5]Department of Biological Sciences, Faculty of Science and Technology, University of Bergen, Bergen, Norway

[6]Department of Paediatrics, Haydom Lutheran Hospital, Mbulu, Manyara, Tanzania

[7]Haydom Global Health Research Centre, Haydom Lutheran Hospital, Haydom, Tanzania

[8]Department of Clinical Science, University of Bergen, Bergen, Norway

[9]Department of Tropical Disease Biology, Liverpool School of Tropical Medicine, Liverpool, UK

[10]National Centre for Tropical Infectious Diseases, Haukeland University Hospital, Bergen, Norway

[11]Norwegian Institute of Public Health, Oslo, Norway

[12]Department of Pediatrics, University Hospital of North Norway, Tromsø, Norway

[13]Lead contact

*Correspondence: veronika.k.pettersen@uit.no

https://doi.org/10.1016/j.xcrm.2026.102752

SUMMARY

Early-life probiotics that strengthen gut resilience in infants are a promising strategy to combat the global emergency of antibiotic resistance. Still, their effects on antibiotic-resistant opportunistic pathogens, i.e., pathobionts, remain unclear. We evaluate the effects of probiotic supplementation in 152 full-term Tanzanian infants enrolled in the ProRIDE trial. Oral probiotics during the first 4 weeks of life increase gut colonization by *Bifidobacterium* species, while suppressing pathobionts, including extended-spectrum β-lactamase-producing *Enterobacterales* (ESBL-E). Integrated metagenomics and metabolomics show that probiotics reduce resistome load and mobilome richness at 6 weeks, accompanied by concurrent shifts in the fecal metabolome. Specifically, the intervention increases lactate and pyruvate and reduces cross-feeding pathways that lead to propionate and butyrate, which partly explains the reduction in ESBL-E carriage. Our study documents putative pathways by which probiotic-driven *Bifidobacterium* colonization modulates the infant gut toward a lower level of antibiotic resistance.

INTRODUCTION

Antimicrobial resistance (AMR) is a persistent threat to global health. As a prime example, systemic infections caused by extended-spectrum β-lactamase-producing *Enterobacterales* (ESBL-E) are not covered by empiric antibiotic regimes and lead to high mortality rates in low- and middle-income countries (LMICs).[1,2] In Tanzania, bloodstream infections with ESBL-E are associated with a case fatality rate as high as 70%, compared to ≈30% for infections caused by susceptible strains,[3,4] underscoring the need for preventive strategies.

The gut microbiome is a first line of defense against colonization by opportunistic pathogens, i.e., pathobionts, including AMR bacteria.[5] As a result, probiotic supplementation has been explored as a tool to reinforce colonization resistance during infancy.[5–7] *Bifidobacterium* species, abundant in breastfed infants, are key bacterial symbionts that support immune development and outcompete taxa with pathogenic potential.[8] Beyond taxonomy, the microbiome harbors a dynamic resistome (the repertoire of antibiotic resistance genes [ARGs]) and mobilome (e.g., plasmids and transposons).[9] These genetic elements are vehicles for the dissemination of resistance, particularly in contexts of antimicrobial exposure.[10] While studies have linked them to clinical and ecological factors (e.g., hospital environments,[11] maternal transmission[12]), the influence of probiotics on the infant resistome and mobilome is still limited, especially in full-term infants from LMICs. Similarly, the relationship between the gut resistome and fecal

© 2026 The Author(s). Published by Elsevier Inc.
This is an open access article under the CC BY license (http://creativecommons.org/licenses/by/4.0/).

metabolome, which could enable its diagnostic use, is currently unknown.

Here, we leverage data from the ProRIDE randomized controlled trial (RCT) conducted at the Haydom Lutheran Hospital,[13,14] Tanzania, representing rural African settings with a high burden of early-life infections and limited RCT evidence. ProRIDE enrolled 2,000 term-born, healthy newborns to investigate the effects of home administration of a multistrain probiotic in the first 4 weeks of life on health outcomes and the gut microbiome.[15] The ProRIDE trial did not show a reduction in the primary outcome (rate of death or hospitalization up to 6 months).[15] However, we observed that a secondary outcome of the trial was a reduction in ESBL-E gut colonization in the probiotic group (164/908, 18%) compared with placebo (206/914, 23%) at 6 weeks, 2 weeks after the supplementation was stopped.[15]

Within this framework, this microbiome sub-study aimed to determine how colonization with the probiotic strains altered gut community composition and reshaped the resistome, mobilome, and metabolome, thereby providing insights into microbiome-targeted strategies for early-life AMR prevention.

RESULTS

Stability of probiotic species colonization in the infant gut

We analyzed 152 infants from the ProRIDE trial (Table S1) with paired metagenomics and metabolomics data (Table S2). We first evaluated gut colonization by the supplemented probiotic strains. Although *Lactobacillus acidophilus* was among the probiotic strains, the species was not detected in metagenomes. On the other hand, *Bifidobacterium longum* subsp. *infantis* Bi-26 has a public genome,[16] enabling strain detection (Figure S1). At 6 weeks, Bi-26 dominated in the probiotic group, comprising 60.3% of *B. longum*-assigned reads (Table S3), which constituted 65.5% and 78.3% of the total bacteria (mean and median, respectively). In the placebo group, *B. longum* represented 38.8% and 10.6% of total bacteria (mean and median, respectively). By 6 months, strain profiles converged across groups, with reads mapping predominantly to a *B. longum* reference genome hadza_2261_Bf14, indicating a closely related background strain in both arms. Bi-26 was absent in the placebo arm, supporting that the *B. longum* signal in the probiotic group largely reflects the probiotic strain.

Probiotic supplementation significantly reshaped gut bacterial composition at 6 weeks of age, 2 weeks after the end of supplementation, but only marginally at 6 months (Figures 1A and S1; Table S4). At 6 weeks, the probiotic group had higher relative abundances of *B. longum*, *Bifidobacterium bifidum*, and *Morganella morganii* compared to the placebo group (ANCOM-BC2, Benjamini-Hochberg adjusted $p < 0.01$; Figure 1B; Table S5). Conversely, pathobionts *Klebsiella pneumoniae*, *Enterococcus faecium*, *Klebsiella oxytoca*, and *Clostridium perfringens* were significantly reduced in the probiotic group (ANCOM-BC2; Benjamini-Hochberg adjusted $p < 0.05$, Figure 1B). In addition to metagenomic profiling, we assessed ESBL-E carriage using culture-based detection. ESBL-E gut colonization was less frequent in the probiotic group at 6 weeks (5% vs. 25%; risk difference [RD], −0.20; 95% confidence interval [CI], −0.319, −0.09; $p = 0.00048$) but no longer significant at 6 months (15% vs. 25%; RD, −0.10; 95% CI, −0.23, 0.03; $p = 0.154$) (Table S1).

We next looked at absolute abundance data (Table S6), which were derived by normalizing each species' read count to the read count of a spike-in control and scaling by the known spike-in cell input. *B. longum* and *B. bifidum* showed significantly higher estimated absolute abundance in the probiotic group at 6 weeks (MaAsLin 3,[17] model accounting for sex, trial arm, place of birth, and antibiotic exposure; Benjamini-Hochberg-adjusted $p < 0.01$; Table S7), consistent with the relative-abundance results. The absolute abundance data were not significantly different for any other species.

Because relative- and absolute-abundance analyses can yield different results when between-group differences reflect taxon detection (presence/absence) rather than changes in abundance among detected taxa, we conducted a prevalence analysis to compare detection frequencies between groups. Three taxa, *Klebsiella variicola*, *Negativicoccus succinicivorans*, and *Erysipelatoclostridium ramosum*, showed significantly lower detection in the probiotic arm (two-sided Fisher's exact test, Benjamini-Hochberg false discovery rate [FDR] correction, Table S8) at 6 weeks. These taxa were not among those identified as differentially abundant in either relative- or absolute-abundance analyses; the prevalence findings, therefore, provide additional context for interpreting the abundance analyses. Of these taxa, *K. variicola* from the *Enterobacterales* order has documented pathogenic potential,[18] whereas *N. succinicivorans* and *E. ramosum* are not canonical early-life pathobionts. Three other pathobionts had lower detection rates in the probiotic arm: *K. oxytoca* (17.7% vs. 1.6%), *E. faecium* (40.3% vs. 25.4%), and *C. perfringens* (16.1% vs. 12.7%). However, the latter results were not FDR significant at 6 weeks ($q \approx 0.28$–0.31) and further attenuated by 6 months.

We also inferred microbial growth dynamics by replication rate analysis using the CoPTR tool, which calculates the ratio of sequencing coverage at the replication origin to that at the replication terminus in a bacterial species[19] (Table S9). Probiotic supplementation appeared to significantly increase growth rates of *B. longum* and *B. bifidum* (Mann-Whitney U test, $p < 0.0001$), while decreasing the growth of *Escherichia coli* at 6 weeks ($p < 0.029$; Figure 1C).

Probiotics also affected the broader community structure. At 6 weeks, the probiotic group had lower bacterial richness (negative binomial model; $p < 0.01$; Figures 1D and S1) and a distinct β-diversity profile (PERMANOVA: Bray-Curtis distance; $R^2 = 16.11\%$; $p < 0.01$; Figure 1E). In addition, α-diversity assessed using the Shannon index was significantly lower in the probiotic group at 6 weeks (Figure S1; Mann-Whitney U test, $p < 0.01$). While observed species richness and β-diversity were no longer significantly different between groups at 6 months (Figure S1), the Shannon index remained significantly lower in the probiotic group (Figure S1; Mann-Whitney U test, $p < 0.01$), indicating persistent differences in community evenness.

Probiotic-mediated reduction of pathobionts

We next examined whether *B. longum* abundance correlated with pathobiont richness, using 18 bacterial species with

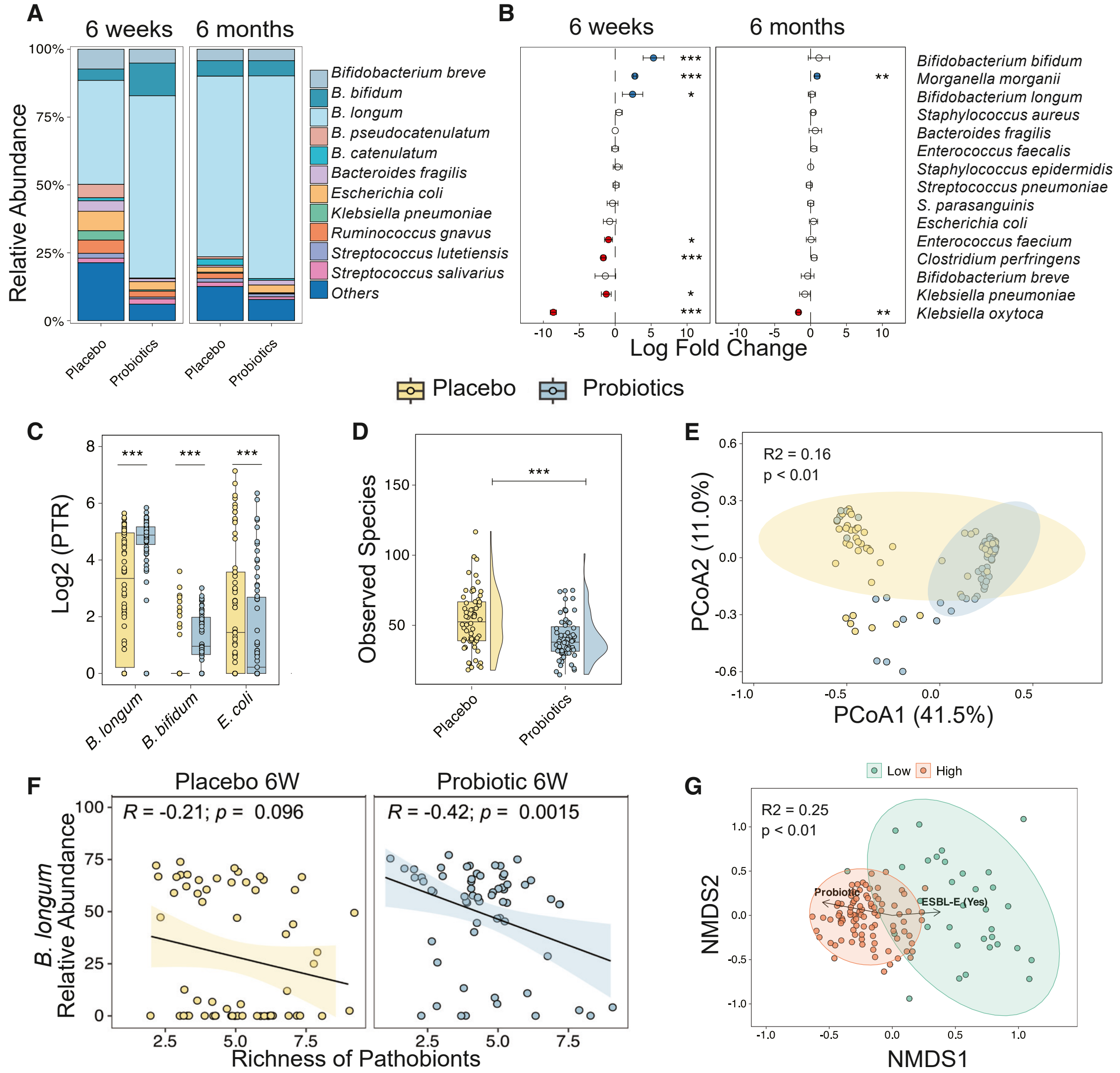

Figure 1. Impact of oral probiotics on bacterial composition of infant gut

(A) Relative abundance of bacterial species in the placebo and probiotic groups, inferred by MetaPhlAn4.

(B) Differential abundance of bacterial species comparing the probiotic vs. placebo groups at 6 weeks and 6 months, identified using ANCOM-BC2 (Benjamini-Hochberg-adjusted *p* values). Effect sizes represent log-fold changes (probiotic relative to placebo); positive values (red) indicate enrichment in the probiotic group, and negative values (blue) indicate enrichment in the placebo group. Filled circles indicate statistically significant effects, while open circles non-significant results.

(C) Replication rate analysis based on Compute Peak-to-Trough Ratio (CoPTR). CoPTR estimates bacterial replication from the ratio of sequencing coverage near the replication origin vs. terminus; higher CoPTR values indicate higher replication activity. Log2-transformed CoPTR values are shown for the probiotic (blue) and placebo (yellow) groups; *p* values computed using the Mann-Whitney U test.

(D) Comparison of observed species richness between groups, with each point representing a sample. *p* values computed using a negative binomial model.

(E) Principal-coordinate analysis (PCoA) illustrating differences in the groups' gut microbiota. Each point represents the mean Bray-Curtis dissimilarity of a sample to all other samples within the same group.

(F) Spearman correlation analysis between *B. longum* relative abundance and the richness of pathobionts (i.e., the number of pathogenic species detected per sample) at 6 weeks.

(G) NMDS and PERMANOVA illustrating dissimilarities between *Bifidobacterium* High and Low clusters at the 6-week time point, with a biplot showing the main variables associated with these clusters.

Statistical significance: $p < 0.05$. ***$p < 0.001$. Metagenomic sequencing was performed once per sample, without technical replicates. Analyses were performed on metagenomic samples from 152 infants (probiotic, $n = 80$; placebo, $n = 72$).

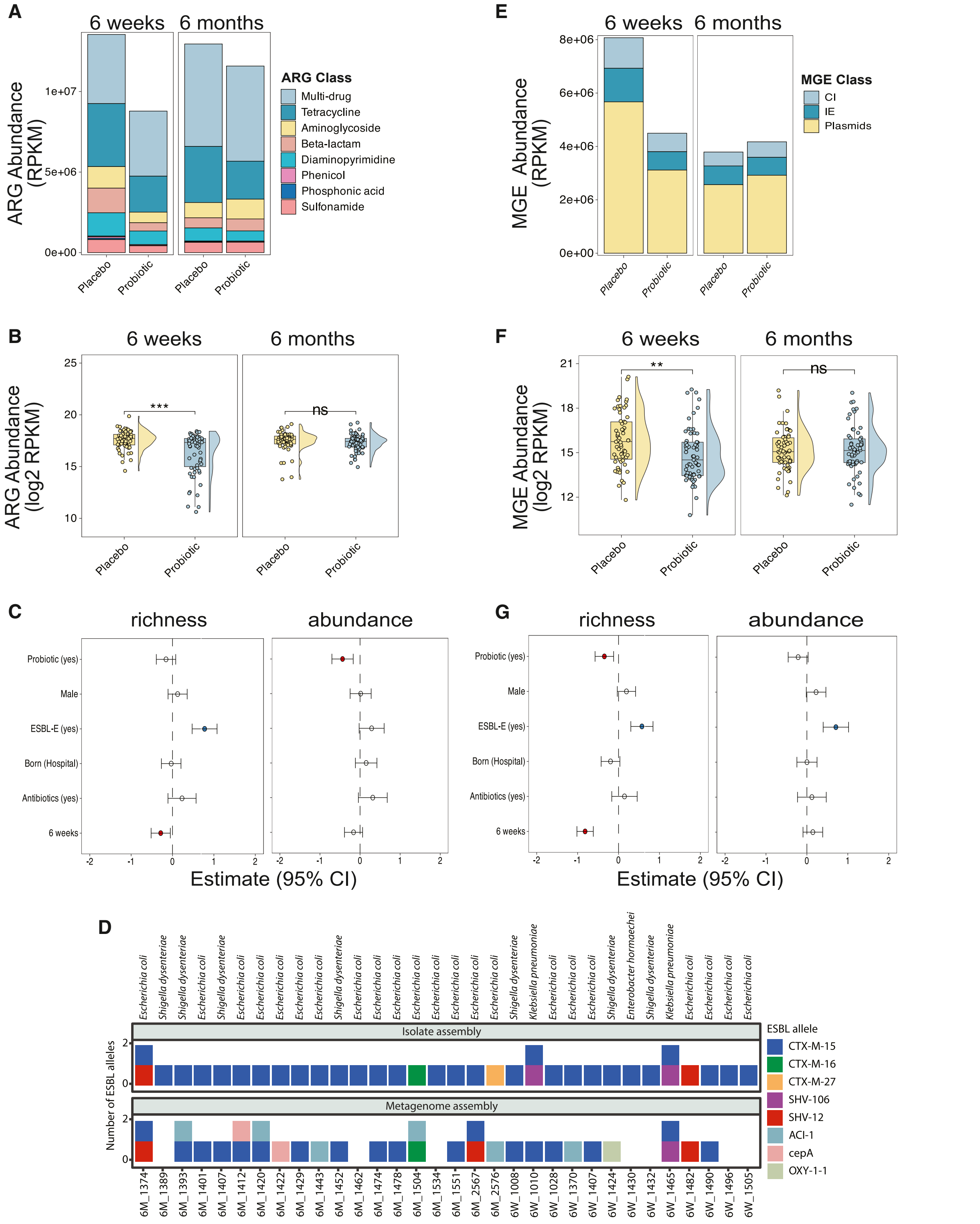

Figure 2. Impact of probiotics on the gut resistome and mobilome

(A) Relative abundance of antibiotic resistance genes (ARGs) in reads per kilobase per million mapped reads (RPKM), stratified by ARG classes (8 most abundant classes). Multidrug-resistant class refers to ARGs linked to resistance to ≥2 antibiotic drugs.

(B) Comparison of ARG abundance between groups, each point representing a sample.

(legend continued on next page)

established pathogenic potential (Table S10).[20–22] In the probiotic group at 6 weeks, *B. longum* abundance showed a moderate negative correlation with pathobiont richness (R = 0.42; $p < 0.01$; Figure 1F). We then applied unsupervised k-means clustering of metagenomic samples to identify co-occurring bacteria. This analysis, based on the abundance of the *Bifidobacterium* genus, identified two clusters: "Low," characterized by a lower abundance of *Bifidobacterium* species (spp.) and a higher abundance of pathobionts, and "High," defined by a higher abundance of *Bifidobacterium* spp. and a lower abundance of pathobionts (Figure S2A). The probiotic group was significantly more likely to belong to the High cluster than the placebo group at 6 weeks ($p < 0.01$, Fisher's exact test, Figure S2B).

To identify factors correlated with the bacterial composition of the two clusters, we performed the Envfit analysis on the non-metric multidimensional scaling (NMDS) ordination (Figure S2C). The tested variables included trial arm, sex, hospitalization, place of birth, antibiotic use, and detection of ESBL-E carriage by culture (Table S2). We were unable to test the impact of feeding type because the vast majority of the included infants were breastfed (148/152; 97%). At 6 weeks, probiotic supplementation emerged as the strongest explanatory factor for microbiota composition, but it explained only a modest proportion of the variance ($R^2 = 0.194$; $p < 0.01$), followed by ESBL-E carriage ($R^2 = 0.086$; $p < 0.01$). By 6 months, ESBL-E carriage was the only factor remaining significantly associated with residual variation in cluster composition ($R^2 = 0.084$; $p < 0.01$), indicating a small but statistically significant contribution relative to the other covariates tested. Consistent with these findings, "Low" and "High" clusters showed a clear separation in ordination analysis (NMDS based on Bray-Curtis dissimilarity and PERMANOVA, $R^2 = 0.25$; $p < 0.01$, Figure 1G). Probiotic supplementation was associated with the "High" cluster, while ESBL-E carriage was linked to the "Low" cluster, and their obtuse angle in the ordination plot indicated a negative correlation between these factors. These results were validated by a generalized linear model, which showed a significant negative association between probiotic supplementation and ESBL-E carriage at 6 weeks ($p < 0.001$; Figure S3A). A random forest model further identified probiotic supplementation as the most important predictor of ESBL-E carriage at 6 weeks (Figure S3B).

Impact of probiotics on the gut resistome and mobilome

Next, we assessed the impact of probiotic supplementation on the resistome. We analyzed metagenomic data against the CARD database, focusing on resistome profiles independently of microbiota composition. We initially identified 1,214 ARGs, which were filtered to 919 more clinically relevant ones that confer resistance to 15 antibiotic classes (Table S11). At 6 weeks, dominant ARGs in the placebo group were linked to multidrug resistance (i.e., conferring resistance to two or more antibiotic drugs, excluding genes encoding efflux pumps), tetracyclines, aminoglycosides, beta-lactams, and diaminopyrimidines (trimethoprim) (Figure 2A). In the probiotic group, the distribution of ARG classes was similar to that in the placebo group at both time points, but their relative abundance (log_2RPKM) was significantly lower at 6 weeks of age (Mann-Whitney U test; $p < 0.01$; Figure 2B).

Using regression models, we determined that ESBL-E carriage was significantly associated with higher ARG richness, whereas probiotic supplementation was associated with lower ARG abundance (Figure 2C, $p < 0.001$). Estimated marginal means (EMMs) analysis of probiotic impact on resistome richness and relative abundance at 6 weeks of age further validated this effect ($p = 0.0316$ and $p < 0.001$, respectively; Figure S4A). We did not detect any change in resistome richness or abundance between the groups at 6-month time point. However, when we extended the resistome analysis to the *Bifidobacterium* "Low" and "High" clusters (defined based on *Bifidobacterium* genus relative abundance), the "Low" cluster had a higher resistome relative abundance (Mann-Whitney U test; $p = 0.0261$; Figure S4B). Last, ordination analysis using Jaccard distances revealed a distinct resistome composition between the groups at 6 weeks (PERMANOVA: $R^2 = 7.23\%$; $p < 0.01$, Figure S4C).

In addition to culture-based detection of ESBL-E carriage, we sequenced ESBL-E isolates from a subset of ESBL-positive samples. To validate the bacterial hosts of detected ARGs, we linked ESBL-E isolate genomes to metagenomic data. We selected 32 metagenome-isolate pairs and compared 33 ARGs from these isolates with the matching metagenomic assemblies (Table S12). Re-analysis of the probiotic impact on resistome using only these isolate-linked ARGs showed the same trend as when using all metagenomic ARGs (Figure S4). Furthermore, taxonomic annotation of ESBL-harboring contigs agreed with the cultured isolate species, and comparison of ESBL genes showed 63% concordance between the isolate and metagenome assemblies (Figure 2D). The remaining discrepancies are likely due to limited sensitivity of metagenome assembly for low-abundance or plasmid-borne genes.

The mobilome facilitates the horizontal transfer of ARGs between bacterial cells. We analyzed the mobilome by querying the metagenomic data against the MobileOG database[23]

(C) Effect of covariates on ARG richness and abundance as estimated by regression models. The *x* axis shows the estimated effect size (with 95% confidence interval); estimates >0 indicate higher richness/abundance and estimates <0 indicate lower richness/abundance relative to the reference level for each covariate. The *y* axis lists the included covariates. ARG richness was modeled using negative binomial regression and ARG abundance using a linear mixed-effects model. Filled circles indicate statistically significant effects (red = positive; blue = negative); open circles indicate non-significant effects.
(D) ESBL genes in isolate and metagenome assemblies. Stacked bar plots show the presence and number of ESBL genes detected in each sample using two approaches: long-read assemblies of 32 ESBL-positive isolates (top) and short-read metagenome assemblies (bottom) of samples from the same individual and time point.
(E) Relative abundance of MGEs in RPKM, stratified by MGE classes. CI, composite integrons; IE, insertion elements.
(F) Comparison of MGE relative abundance between groups, each point representing a sample.
(G) Effect of variables on mobilome richness and abundance (95% confidence interval). Richness was modeled with negative binomial regression and abundance with LMM. Statistical significance: $p < 0.05$ (Mann-Whitney U test). ***$p < 0.001$; **$p < 0.01$. Resistome and mobilome analyses were performed on metagenomic samples from 152 infants (probiotic $n = 80$; placebo $n = 72$).

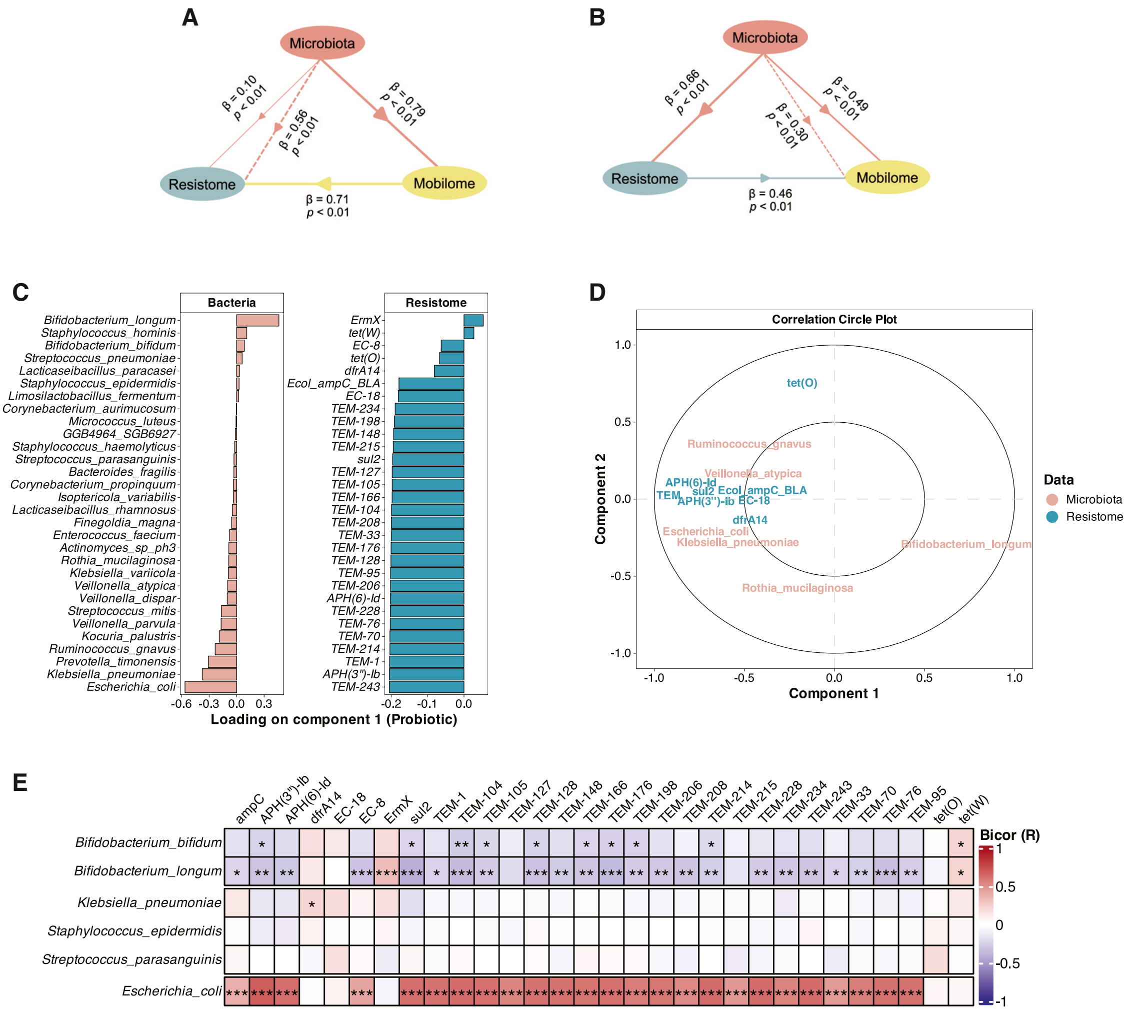

Figure 3. Interactions between microbiota, resistome, and mobilome at 6 weeks

(A and B) Partial least squares path modeling illustrates interactions between microbiota, resistome, and mobilome, with (A) model on the right set to use mobilome data to predict interactions with resistome and (B) model on the left using resistome data to predict interactions with mobilome.

(C) Top 30 discriminative predictors identified by DIABLO on component 1 separating probiotic and placebo groups. Bars show the loadings for bacterial species (left) and ARGs (right); negative loadings indicate higher relative abundance in the placebo group, whereas positive loadings indicate higher abundance in the probiotic group.

(D) Correlation circle plot illustrating relationships between bacterial species and ARGs.

(E) Correlation between selected pathobionts and probiotic species, determined using biweight midcorrelation. Statistical significance: $p < 0.05$. *$p < 0.05$; **$p < 0.01$; ***$p < 0.001$. Analyses were restricted to 6-week samples (probiotic $n = 80$; placebo $n = 72$).

(Table S13). Plasmids were the most abundant mobile genetic elements (MGEs) (Figure 2E), and the mobilome relative abundance was significantly lower in the probiotic group compared to the placebo group at 6 weeks of age (Mann-Whitney U test; $p < 0.01$; Figure 2F), while ESBL-E carriage was significantly associated with higher mobilome richness and overall load (Figure 2G, $p < 0.001$). Complementary EMM analysis revealed a significant reduction in mobilome richness and load at 6 weeks of age ($p < 0.01$ and $p < 0.02$, respectively; Figure S5). Finally, β-diversity analysis using Jaccard distances revealed distinct mobilome composition between the probiotic and placebo groups at 6 weeks (PERMANOVA: $R^2 = 2.5\%$; 999 permutations; $p < 0.01$). None of these associations was present at 6 months of age.

Interactions between bacterial microbiota, ARGs, and MGEs

Given the similarities among microbiota, resistome, and mobilome profiles (Figure S6), we employed partial least squares path modeling to describe interaction pathways among bacterial composition, ARGs, and MGEs. We assumed that microbiota affects both resistome and mobilome and estimated the correlation between them. In the first model (Figure 3A), which estimated the influence of mobilome on resistome, bacterial microbiota had a strong direct effect on MGEs (path coefficient $\beta = 0.79$, $p < 0.01$) and a weak direct effect on ARG composition ($\beta = 0.10$, $p < 0.01$). At the same time, microbiota exerted a strong indirect effect on ARGs via MGEs ($\beta = 0.56$, $p < 0.01$) and MGEs

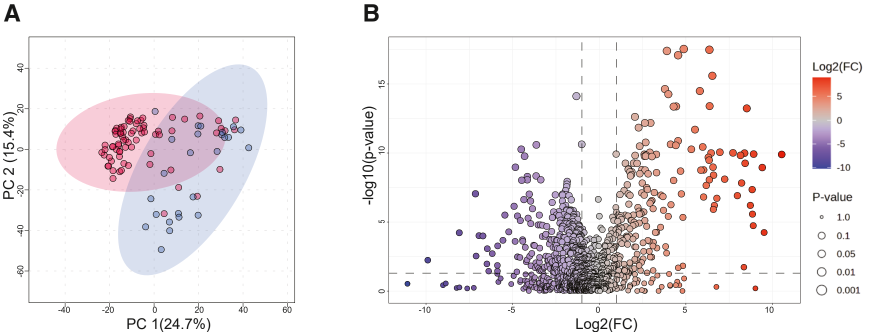

Figure 4. Untargeted metabolomics of infant stool samples from 6 weeks

(A) Principal-component analysis of "High" (red) and "Low" (blue) clusters based on *Bifidobacterium* spp. abundance (Euclidean distance, PERMANOVA, $R^2 = 0.25$, $p = 0.001$).

(B) Statistical comparison of the High and Low clusters' metabolic profiles visualized as a volcano plot (Mann-Whitney U test, $p < 0.05$, FDR, 2-fold change threshold, unequal group variance). In the High cluster, 354 and 190 features were significantly decreased and increased, respectively, compared to the Low cluster. Untargeted metabolomics was performed on 200 samples from 101 infants. Each point represents one biological sample; analyses were conducted in a single analytical run without technical replication.

exerted a strong direct effect on ARG composition ($\beta = 0.71$, $p < 0.01$). To explore whether the resistome also impacts MGE composition, we modified the model (Figure 3B) by allowing resistome data to predict interactions with mobilome. This adjustment revealed a strong direct effect of ARGs on MGEs ($\beta = 0.46$, $p < 0.01$). In this model, microbiota was a strong predictor of both MGEs ($\beta = 0.49$, $p < 0.01$) and ARGs ($\beta = 0.66$, $p < 0.01$) and had a moderate indirect effect on MGEs through ARGs ($\beta = 0.30$, $p < 0.01$). Altogether, these models can be interpreted as follows: (1) microbiota influences ARG composition through MGEs and (2) MGE composition contributes to ARG profile, reinforcing their interconnectedness.

We then predicted bacterial hosts of ARGs by data integration analysis for biomarker discovery using latent components (DIABLO). Among the top 30 discriminative predictors, we observed that the relative abundance of several pathobionts and ARGs was associated with the placebo group (Figure 3C). In contrast, commensal bacteria, such as *B. longum*, were associated with the probiotic group. Additionally, correlation circle plot revealed strong negative correlations between *B. longum* and key pathobionts, *E. coli*, and *K. pneumoniae* (Figure 3D), as well as between *B. longum* and the most variable ARGs. In contrast, *E. coli* and *K. pneumoniae* exhibited strong positive correlations with the resistome, consistent with our previous findings, which describe them as major ARG reservoirs.[24,25] To validate these predictions, we performed a biweight midcorrelation analysis and filtered the results to retain 18 selected bacterial species with pathogenic potential[20–22] and probiotic species. Features with low variance or missing values that resulted in undefined correlations were excluded from the analysis. The results aligned with the DIABLO output: *E. coli* showed a strong positive correlation with the top 30 most variable ARGs, whereas *B. longum* had a strong negative correlation (Figure 3E).

Impact of probiotic supplementation on fecal metabolome

To assess functional changes in the gut microbiome, we performed untargeted metabolomics on 200 stool samples from 101 infants (Table S2). As with the metagenomic data, no significant group-level differences were detected at 6 months of age. At 6 weeks, principal-component analysis revealed distinct metabolite profiles between the groups (Euclidean distance, PERMANOVA, $R^2 = 0.07$, $p = 0.004$, Figure S7A). We detected 45 significantly increased and 37 decreased metabolic features in the probiotic group (Mann-Whitney U test, $p < 0.05$, FDR correction, Figure S7B). Because the supplementation strongly increased *Bifidobacterium* abundance, we additionally stratified 6-week samples by *Bifidobacterium* spp. levels. The "High" and "Low" groups formed well-separated groups (Euclidean distance, PERMANOVA, R^2 0.25, $p = 0.001$, Figure 4A). Infant samples from the High cluster showed 190 significantly increased and 354 decreased metabolite features (Mann-Whitney U test, $p < 0.05$, FDR, Figure 4B), supporting a broad metabolic impact of *Bifidobacterium* colonization.

Closer examination revealed that metabolites enriched in the High cluster were putatively annotated as simple sugars and derivatives, with hexitol and a disaccharide containing two galactopyranoses annotated with high confidence (scores 0.518 and 0.955, respectively, Figure S8). Metabolite features with significantly decreased abundance were putatively annotated as lipid-like compounds, amino acid residues, di- and oligosaccharides, and bile acids. Notably, the bile acid cholate was annotated with high confidence (score 0.976) and was among the most abundant metabolites across samples. These results link probiotic supplementation to alterations in both microbial and host metabolites. Finally, to confirm pathway abundance in the metagenomic data, we performed functional profiling using HUMAnN3.[26] Both community-level and species-resolved pathways differed between the High and Low clusters, with enrichment of *Bifidobacterium*-associated primary fermentation and amino acid biosynthesis pathways in the High cluster (Mann-Whitney U test, $p < 0.05$, FDR, Table S14).

Bacterial metabolites predictive of ESBL-E carriage

Given the impact of probiotics on the metabolome, we asked whether specific bacterial metabolites contribute to pathobiont suppression and reduced ARG load. Using targeted metabolomics, we quantified 23 key bacterial metabolic intermediates and end products (15 short-chain fatty acids [SCFAs] and 8 organic acids [OAs]; Table S15). A comparison of these metabolites in 241 stool samples from 152 infants obtained at 6 weeks (Table S2) revealed significantly higher levels of citrate, pyruvate, and lactate in the probiotic group and significantly higher propionate levels in the placebo group at 6 weeks (linear regression

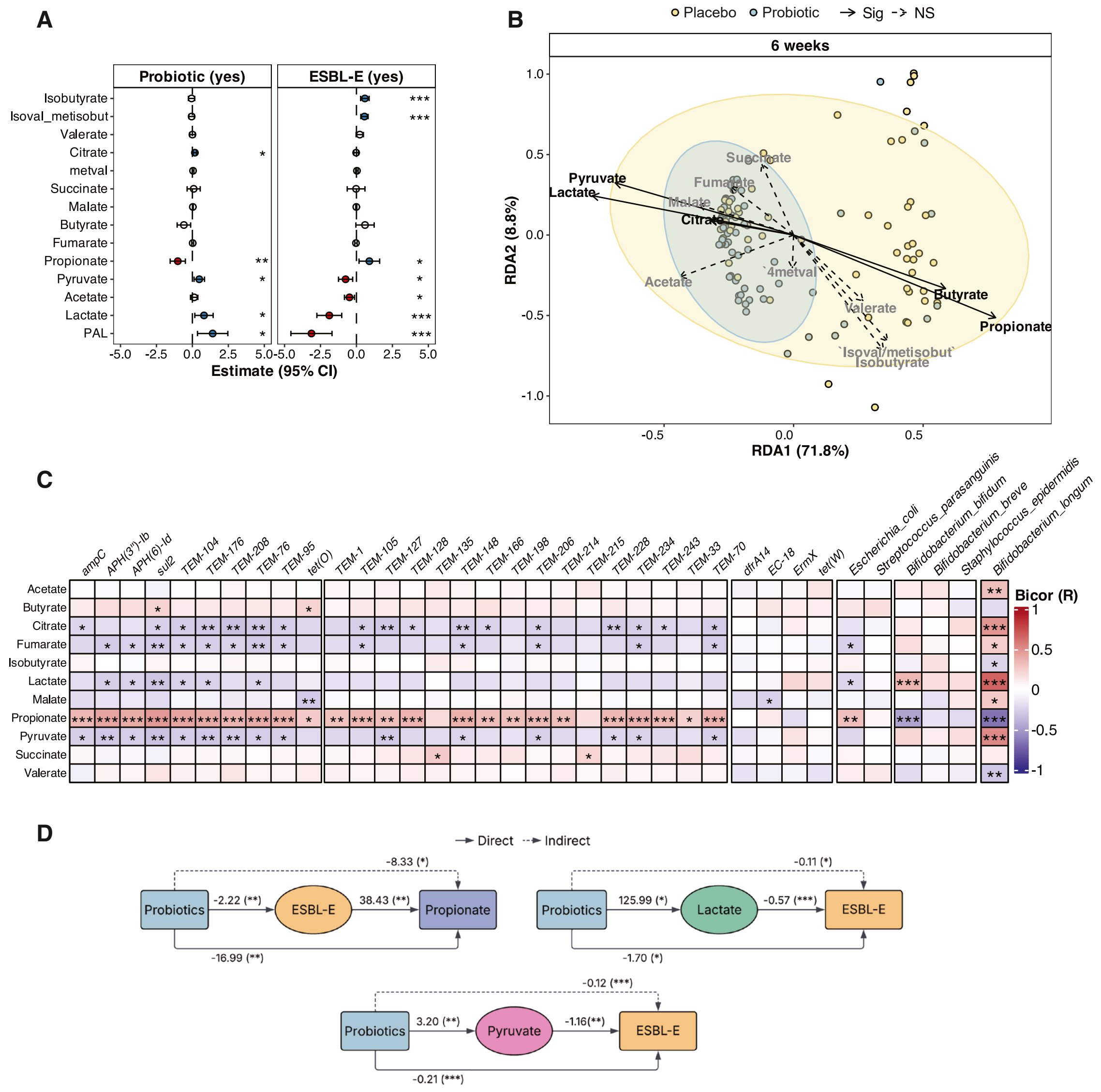

Figure 5. Targeted metabolomics of infant stool samples from 6 weeks

(A) Differential abundance of fecal metabolites at 6 weeks estimated using multivariable linear models (Benjamini-Hochberg-adjusted *p* values). Dots represent regression coefficients with 95% confidence intervals for the association with probiotic supplementation (left) and ESBL-E carriage (right). Filled circles indicate statistically significant effects (red = positive; blue = negative), while open circles non-significant results. PAL, combined output of pyruvate, acetate, and lactate.

(B and C) Redundancy discriminant analysis (RDA) biplot depicting the relationship between gut bacterial communities and SCFAs. Each point represents an infant stool sample, colored by trial arm (probiotic vs. placebo), while arrows represent SCFAs with significant loadings in the RDA (permutation test, $p < 0.05$). The direction and length of each arrow indicate the strength and direction of association between a given SCFA and the microbiota profiles in each group. (C) Biweight midcorrelation analysis showing correlations between metabolites and the top 30 most variable ARGs.

(D) Three mediation analysis models testing the relationships between probiotics, ESBL-E colonization, and the metabolites propionate, lactate, and pyruvate. For propionate, the model evaluates whether the effect of probiotics on propionate levels is mediated by ESBL-E. For lactate and pyruvate, the models test whether the effect of probiotics on ESBL-E colonization is mediated by each metabolite. Dotted lines indicate indirect effects; full lines show direct interactions. Positive and negative numbers indicate positive and negative directions of the effects. Statistical significance: $p < 0.05$. *$p < 0.05$; **$p < 0.01$; *** $p < 0.001$. Targeted metabolomics included 241 samples from 152 infants at 6 weeks. Each point represents one biological sample.

model with the Benjamini-Hochberg correction, Figure 5A). In addition, several SCFAs (isobutyrate, isovalerate/methyl isobutyrate, and propionate) were positively associated with ESBL-E carriage, while pyruvate, acetate, and lactate showed negative associations. Similarly, in clusters defined by *Bifidobacterium* spp. abundance, levels of lactate, pyruvate, butyrate, and propionate were significantly different between the High and Low clusters (Figure S9A).

In infancy, primary fermenters such as *Bifidobacterium* spp., produce acetate and lactate through the Bifid shunt (fructose-6-phosphate pathway),[27] whereas secondary fermenters generate propionate and butyrate through cross-feeding on

lactate, acetate, and succinate.[28] To capture metabolites involved in the Bifid shunt, we combined the abundances of pyruvate, acetate, and lactate (PAL, Figure 5A). This combination further strengthened the positive association with the probiotic group. Next, we performed redundancy discriminant analysis to investigate how SCFAs and OAs relate to the overall microbiota. Citrate, pyruvate, and lactate were significantly associated with microbiota composition in the probiotic group, whereas propionate and butyrate were strongly associated with the microbiota profiles in the placebo group (Figure 5B). These patterns were supported by a random forest model, where propionate, lactate, citrate, and pyruvate emerged as the top predictors of microbiota composition (Figure S9B), as well as with DIABLO, which identified lactate and pyruvate as the top predictors of the probiotic group and propionate as a predictor of the placebo group (Figure S9C). Biweight midcorrelation analysis further showed that acetate, citrate, lactate, and pyruvate were significantly associated with *B. longum*, while propionate correlated with *E. coli* and many of the top 30 variable ARGs, particularly bla_{TEM}. In contrast, citrate, fumarate, lactate, and pyruvate were significantly negatively associated with these ARGs (Figure 5C).

Propionate, pyruvate, and lactate were each associated with both probiotic supplementation and ESBL-E carriage. To test the hypothesis that these metabolites mediate the association between probiotic administration and reduced ESBL-E carriage, we conducted an exploratory mediation analysis. This suggested that probiotics may indirectly reduce propionate levels by decreasing ESBL-E carriage, while increases in lactate and pyruvate may directly mediate the effect of probiotics on ESBL-E colonization (Figure 5D).

Reinforcement of *Bifidobacterium* metabolic signatures by probiotic supplementation

To further assess the metabolite dynamics, we examined indicators of metabolic fluxes, i.e., ratios and correlations among individual SCFAs and OAs. At 6 weeks, infants with High *Bifidobacterium* spp. abundance in both the probiotic and placebo groups showed dominance of lactate and acetate, with lower levels of other SCFAs and OAs. Low *Bifidobacterium* groups had a more diverse profile, with higher proportions of succinate, propionate, and butyrate (Figure S10A), consistent with activity of secondary cross-feeding fermenters.

In the High *Bifidobacterium* groups, lactate and acetate were positively correlated (probiotic group, Spearman coefficient $R = 0.66$; $p < 0.001$; placebo group, $R = 0.18$; $p < 0.001$, Figure S10B), in line with co-production via the Bifid shunt. On the other hand, the 6-week placebo Low *Bifidobacterium* group showed a significant negative correlation between succinate and propionate ($R = -0.47$; $p < 0.01$, Figure S10C), indicating conversion via the succinate pathway, a route for propionate production.[29] This metabolite flux was not significant in the other groups.

Additional differences emerged between the placebo and probiotic groups. The placebo High *Bifidobacterium* group showed significant negative correlations between several SCFAs (propionate, butyrate, isobutyrate, valerate, and isovalerate/methyl isobutyrate) and OAs (citrate, fumarate, pyruvate, malate, succinate, and lactate), suggesting SCFA production via these OA intermediates. This pattern was attenuated in the probiotic High group (Figure S10B), despite similar metabolite abundances.

DISCUSSION

This ancillary analysis of the ProRIDE trial examined how a 4-week probiotic supplementation in healthy full-term infants in Tanzania affected pathobiont dynamics and features of the gut microbiome. While clinical outcomes up to 6 months did not differ between arms, probiotic supplementation reduced microbiota diversity, pathobiont abundance, and the replication rate of a key host of ARGs, *E. coli*. Prior work indicates that probiotics inhibit pathobionts by nutrient competition, adhesion-site exclusion, and secretion of antimicrobial compounds and may also modulate host immunity.[30–32] Our data extend these findings by highlighting metabolic activities that may suppress ARG-carrying bacteria.

Patterns of *B. longum* colonization differ across populations, with human milk oligosaccharide (HMO)-adapted *B. longum* subsp. *infantis* being more prevalent in breastfed infants in non-industrialized settings.[33,34] Our strain-level analysis showed that the supplemental Bi-26 strain was dominant at 6 weeks but replaced by a native *B. longum* strain by 6 months, indicating transient engraftment. Similarly, probiotic effects on ARG carriage were transient, with a reduction in ARG burden detectable 2 weeks after the cessation of supplementation, similar to earlier studies.[35–37] Culture-based data indicated that the intervention substantially reduced early-life exposure to ESBL-E, as measured at 6 weeks, even though the point prevalence of ESBL-E carriage and resistome/mobilome loads converged between arms by 6 months. This pattern suggests that probiotics primarily limit early acquisition and expansion of ESBL-E, rather than sustaining long-term differences once the gut microbiota stabilizes. Differences in prevalence, rather than abundance, explained the discrepancies between relative and absolute abundance data, consistent with niche exclusion driven by bifidobacterial expansion. Probiotic supplementation also reduced MGE richness and load, potentially limiting horizontal ARG transfer. While previous work has explored probiotic effects on the infant mobilome,[24,38] data on term infants in LMICs are scarce. Our findings show that reductions in *E. coli* and other ARG pathobionts[24,39–41] likely contributed to the observed decrease in resistome and mobilome, as ARG and MGE maintenance depends on their bacterial hosts.

Metabolomic profiling provided additional insights, consistent with the known metabolic capacities of the supplemented strains.[16,27] Infants receiving probiotics showed higher levels of simple sugars, pyruvate, acetate, and lactate and lower di- and oligosaccharides in stool at 6 weeks. This pattern corroborates a gut ecosystem dominated by *Bifidobacterium* spp., which utilize the Bifid shunt to ferment complex carbohydrates (like HMOs) into acetate and lactate via pyruvate. These simple OAs may lower intestinal pH, thereby suppressing colonization by pH-sensitive bacterial taxa, such as ESBL-E. Mediation analysis supported this hypothesis, suggesting that lactate and pyruvate mediate the probiotic effect on ESBL-E; however, further experimental validation is required to establish causality.

Consistent with our findings, an earlier study in preterm infants reported higher acetate and lower stool pH following probiotic administration[42] and a longitudinal study linked *Enterobacterales*-dominated microbiomes with reduced acetate and increased succinate.[43]

Propionate and butyrate, typically produced by secondary fermenters,[28] were enriched in Low *Bifidobacterium* communities, which also showed higher abundances of *Enterobacterales* and a more diverse metabolic profile, consistent with expanded secondary fermentation. Propionate was positively associated with *E. coli* abundance, ESBL-E carriage, and several ARGs, whereas lactate, pyruvate, acetate, citrate, and fumarate showed negative associations with these ARGs. These findings suggest that propionate and butyrate are markers of a community configuration in which ESBL-E co-occurs with secondary fermenters, rather than direct metabolic products of ESBL-E. While propionate and butyrate are generally linked to gut and host health, especially in older children and adults, their role appears context dependent. In early-life settings, a metabolome dominated by lactate and acetate, driven by bifidobacterial primary fermentation, seems to be associated with lower ESBL-E carriage, in line with evidence that OA-mediated acidification can inhibit antibiotic-resistant Enterobacteriaceae.[44] The relationship between ESBL-E carriage and SCFA has so far been mainly studied in models,[44,45] with an *in vitro* study suggesting that propionate may enhance virulence traits in pathogenic *E. coli*.[46] A propionate-enriched environment might thus be beneficial for the proliferation of drug-resistant pathobionts.

In conclusion, in the ProRIDE cohort of mostly vaginally born and breastfed infants with naturally high *Bifidobacterium* levels, we found a modest reduction in ESBL-E carriage in the probiotic group. WHO has no specific recommendations on probiotic use for term infants in LMICs but has suggested that probiotics can be considered for very preterm infants.[47] Our study documents that probiotics can transiently reshape the infant gut's resistome, mobilome, and metabolome. We propose a model in which probiotic-driven bifidobacterial dominance lowers *E. coli* replication, reduces ARG and MGE carriage, and promotes a lactate/pyruvate/acetate-enriched environment unfavorable to ESBL-E. Due to the transient effects, sustained supplementation or optimized dosing may be needed, and future work should assess the long-term impact of probiotics on ARG transmission to inform AMR prevention in vulnerable populations.

Limitations of the study

We acknowledge several limitations, including the low number of time points for sample collection and the resulting constrained resolution of microbial dynamics. Detailed data on antibiotic use, including type, duration, indication, timing after birth, and maternal intrapartum exposure, were unavailable. Similarly, dietary factors, such as the introduction of solid foods, were not documented.

We used 2 × 150-bp shotgun sequencing, a methodology that can reach species-level resolution, but accuracy drops for recent divergences, species complexes, and novel or under-represented taxa.[48] Nonetheless, our strategy of using deep paired-end data (∼140 M reads/sample) combined with conservative filters and coverage/breadth checks yielded high confidence for abundant, genetically distinct species. We performed taxonomic profiling using MetaPhlAn4 and the CHOCOPhlAn marker-gene database, which provided reliable species-level assignments but insufficient resolution to distinguish *B. longum* subsp. *longum* from *B. longum* subsp. *infantis*. Given the short-read nature of the data, subspecies-level calls were considered unreliable, and we, therefore, limited interpretation to the species level. Similarly, the absence of the probiotic strain *L. acidophilus* in stool metagenomes should be interpreted with caution, as this species typically colonizes at low abundance and may fall below the detection limit of shotgun sequencing, even at high coverage.

Regarding resistome inferences, we relied on a single reference database (CARD), which may influence both the spectrum and absolute number of ARGs detected. CARD is currently the best-curated, frequently updated resource with detailed annotations, but it is largely built from ARGs in culturable and pathogenic bacteria and may under-represent ARGs from non-culturable gut taxa. Other databases differ in scope, update frequency, and clinical focus, so ARG counts may not be directly comparable across studies using alternative resources. While some ARGs represented in other databases may be missing, this is unlikely to affect the relative within-cohort and longitudinal comparisons that underpin our conclusions.

RESOURCE AVAILABILITY

Lead contact

Further information and requests for resources should be directed to the lead contact, Veronika K. Pettersen.

Materials availability

This study did not generate new, unique reagents.

Data and code availability

- Data: Shotgun metagenomic reads (human reads removed) are deposited at the NCBI Sequence Read Archive (SRA) BioProject: PRJNA1305341. Whole-genome sequencing data of ESBL-E isolates are deposited at SRA BioProject : PRJNA1370689. Untargeted metabolomics data are available at MassIVE : MSV000098821. Identified MobileOG marker families and associated annotations are available at Zenodo: https://doi.org/10.5281/zenodo.11491353.
- Code: Custom analysis scripts used for data processing and statistical analyses are available at Zenodo: https://doi.org/10.5281/zenodo.15626017.
- Other: Any additional information required to reanalyze the data reported in this work is available from the lead contact upon request.

ACKNOWLEDGMENTS

We extend our heartfelt thanks to the field workers at Haydom Global Health Research Centre for their dedication and hard work. We extend special gratitude to Ketil Størdal, Blandina Theophil Mmbaga, Charles Makasi, and Sven Gudmund Hinderaker for their oversight of the Data and Safety Monitoring Board; Thomas Eagan for providing randomization support; and Helene Heitmann Sandnes for managing the randomization plan. We also thank AmbioGen for DNA extraction and sequencing, as well as Sigma2 Norwegian Research Infrastructure Service for their computing resources. Above all, we are deeply grateful to the village leaders and families around Haydom Lutheran Hospital for their trust and participation; this study would not have been possible without them. This trial received main funding from Western Norway Regional Health Authority (grant no. 912267), Trond Mohn Foundation (TMS2020TMT11), JPIAMR STRESST project (NFR333432), and Northern

CellPress
OPEN ACCESS

Norway Regional Health Authority (grant no. HNF-1512-20 and HNF1705-24). Additional funding was provided by Center for New Antibacterial Strategies (CANS) through Tromsø Research Foundation to V.K.P. (TFS18_CANS_AS-HVF).

AUTHOR CONTRIBUTIONS

V.K.P. designed this sub-study of the ProRIDE trial, with contributions from C.K. and A.B. N.L., C.K., I.H.L., and V.K.P. secured funding. M.J., S.J.M., and B.B. performed data curation. A.B. crafted the bioinformatics pipeline, conducted analyses, performed statistical and machine-learning analyses, integrated multi-omics data, and visualized data. A.B. also drafted the initial manuscript. G.H.B. carried out metabolomics experiments and data analyses. I.H.L. and M.A.K.H. provided the genomes of the ESBL-E isolates. M.A.K.H. performed the whole-genome bioinformatics and isolate-metagenome ARG linkage analyses. V.K.P. supervised data analyses and manuscript preparation and finalized the figures. All authors provided critical feedback on the manuscript and approved the final version for submission.

DECLARATION OF INTERESTS

The authors declare that they have no competing interests.

DECLARATION OF GENERATIVE AI AND AI-ASSISTED TECHNOLOGIES IN THE WRITING PROCESS

During the preparation of this work, the authors used generative AI-assisted language tools to support editing for clarity, grammar, and language refinement. No AI tools were used for data analysis, data interpretation, figure generation, or scientific decision-making. All content was critically reviewed, revised, and approved by the authors, who take full responsibility for the accuracy and integrity of the manuscript.

STAR★METHODS

Detailed methods are provided in the online version of this paper and include the following:

- KEY RESOURCES TABLE
- EXPERIMENTAL MODEL AND STUDY PARTICIPANT DETAILS
  - Ethics statement
  - Study design and participants
- METHOD DETAILS
  - DNA extraction from human stool samples and sequencing
  - ESBL-E screening
  - Bioinformatics preprocessing of metagenomic data
  - Metagenome, resistome, and mobilome profiling
  - Linking ARGs in metagenomes to ESBL-E isolates
  - Analysis of ESBL genes in isolates and corresponding metagenomes
  - Mass spectrometry (MS)-based metabolomics analyses
  - UPLC-MS/MS analysis for quantification of short-chain fatty acids and organic acids
  - Preparation of external and internal standards and quality controls
  - Targeted UPLC-MS/MS instrumentation and data processing
  - Untargeted LC-MS/MS profiling
  - LC-MS/MS instrumentation and data acquisition
  - Untargeted LC-MS/MS quality control assessment
  - Raw MS/MS data processing
  - SIRIUS-based annotation
  - Feature-based molecular networking and statistics
- QUANTIFICATION AND STATISTICAL ANALYSIS
  - Linear mixed models and differential abundance analyses
  - β-diversity, ordination, and clustering analyses
  - Other statistical analyses and data visualization
  - Statistical analysis of metabolomic data
  - Computational resources

SUPPLEMENTAL INFORMATION

Supplemental information can be found online at https://doi.org/10.1016/j.xcrm.2026.102752.

Received: August 24, 2025
Revised: December 24, 2025
Accepted: March 18, 2026
Published: April 20, 2026

REFERENCES

1. Cocker, D., Sammarro, M., Chidziwisano, K., Elviss, N., Jacob, S.T., Kajumbula, H., Mugisha, L., Musoke, D., Musicha, P., Roberts, A.P., et al. (2022). Drivers of resistance in Uganda and Malawi (DRUM): a protocol for the evaluation of one-health drivers of extended spectrum beta lactamase (ESBL) resistance in low-middle income countries (LMICs). Wellcome Open Res. *7*, 55.
2. Husna, A., Rahman, M.M., Badruzzaman, A.T.M., Sikder, M.H., Islam, M.R., Rahman, M.T., Alam, J., and Ashour, H.M. (2023). Extended-spectrum β-lactamases (ESBL): challenges and opportunities. Biomedicines *11*, 2937.
3. Blomberg, B., Jureen, R., Manji, K.P., Tamim, B.S., Mwakagile, D.S.M., Urassa, W.K., Fataki, M., Msangi, V., Tellevik, M.G., Maselle, S.Y., et al. (2005). High rate of fatal cases of pediatric septicemia caused by gram-negative bacteria with extended-spectrum beta-lactamases in Dar es Salaam, Tanzania. J. Clin. Microbiol. *43*, 745–749.
4. Blomberg, B., Manji, K.P., Urassa, W.K., Tamim, B.S., Mwakagile, D.S.M., Jureen, R., Msangi, V., Tellevik, M.G., Holberg-Petersen, M., Harthug, S., et al. (2007). Antimicrobial resistance predicts death in Tanzanian children with bloodstream infections: a prospective cohort study. BMC Infect. Dis. *7*, 43.
5. Pamer, E.G. (2016). Resurrecting the intestinal microbiota to combat antibiotic-resistant pathogens. Science *352*, 535–538.
6. Casaburi, G., Duar, R.M., Vance, D.P., Mitchell, R., Contreras, L., Frese, S.A., Smilowitz, J.T., and Underwood, M.A. (2019). Early-life gut microbiome modulation reduces the abundance of antibiotic-resistant bacteria. Antimicrob. Resist. Infect. Control *8*, 131.
7. Momo Kadia, B., Otiti, M.I., Ramsteijn, A.S., Sow, D., Faye, B., Heffernan, C., Hall, L.J., Webster, J.P., Walker, A.W., and Allen, S. (2024). Modulating the early-life gut microbiota using pro-pre-and synbiotics to improve gut health, child development, and growth. Nutr. Rev. *82*, 244–247.
8. Arrieta, M.C., Stiemsma, L.T., Amenyogbe, N., Brown, E.M., and Finlay, B. (2014). The intestinal microbiome in early life: health and disease. Front. Immunol. *5*, 427.
9. Thänert, R., Sawhney, S.S., Schwartz, D.J., and Dantas, G. (2022). The resistance within: Antibiotic disruption of the gut microbiome and resistome dynamics in infancy. Cell Host Microbe *30*, 675–683.
10. Vatanen, T., Jabbar, K.S., Ruohtula, T., Honkanen, J., Avila-Pacheco, J., Siljander, H., Stražar, M., Oikarinen, S., Hyöty, H., Ilonen, J., et al. (2022). Mobile genetic elements from the maternal microbiome shape infant gut microbial assembly and metabolism. Cell *185*, 4921–4936.e15.
11. Ravi, A., Estensmo, E.L.F., Abée-Lund, T.M.L., Foley, S.L., Allgaier, B., Martin, C.R., Claud, E.C., and Rudi, K. (2017). Association of the gut microbiota mobilome with hospital location and birth weight in preterm infants. Pediatr. Res. *82*, 829–838.
12. Pärnänen, K., Karkman, A., Hultman, J., Lyra, C., Bengtsson-Palme, J., Larsson, D.G.J., Rautava, S., Isolauri, E., Salminen, S., Kumar, H., et al. (2018). Maternal gut and breast milk microbiota affect infant gut antibiotic resistome and mobile genetic elements. Nat. Commun. *9*, 3891.
13. Kamala, B.A., Ersdal, H.L., Moshiro, R.D., Guga, G., Dalen, I., Kvaløy, J.T., Bundala, F.A., Makuwani, A., Kapologwe, N.A., Mfaume, R.S., et al. (2025). Outcomes of a Program to Reduce Birth-Related Mortality in Tanzania. N. Engl. J. Med. *392*, 1100–1110.

14. Mduma, E.R., Gratz, J., Patil, C., Matson, K., Dakay, M., Liu, S., Pascal, J., McQuillin, L., Mighay, E., Hinken, E., et al. (2014). The etiology, risk factors, and interactions of enteric infections and malnutrition and the consequences for child health and development study (MAL-ED): description of the Tanzanian site. Clin. Infect. Dis. *59*, S325–S330.

15. Klingenberg, C., Justine, M., Moyo, S.J., Löhr, I.H., Gideon, J., Mdoe, P., Mduma, E., Manyahi, J., Bargheet, A., Pettersen, V.K., et al. (2025). Home administration of a multistrain probiotic once per day for 4 weeks to newborn infants in Tanzania (ProRIDE): a double-blind, placebo-controlled randomised trial. Lancet. Glob. Health *13*, e1082–e1090.

16. Zabel, B., Yde, C.C., Roos, P., Marcussen, J., Jensen, H.M., Salli, K., Hirvonen, J., Ouwehand, A.C., and Morovic, W. (2019). Novel Genes and Metabolite Trends in Bifidobacterium longum subsp. infantis Bi-26 Metabolism of Human Milk Oligosaccharide 2′-fucosyllactose. Sci. Rep. *9*, 7983.

17. Nickols, W.A., Kuntz, T., Shen, J., Maharjan, S., Mallick, H., Franzosa, E.A., Thompson, K.N., Nearing, J.T., and Huttenhower, C. (2026). MaAsLin 3: refining and extending generalized multivariable linear models for meta-omic association discovery. Preprint at bioRxiv. https://doi.org/10.1101/2024.12.13.628459.

18. Maatallah, M., Vading, M., Kabir, M.H., Bakhrouf, A., Kalin, M., Nauclér, P., Brisse, S., and Giske, C.G. (2014). Klebsiella variicola Is a Frequent Cause of Bloodstream Infection in the Stockholm Area, and Associated with Higher Mortality Compared to K. pneumoniae. PLoS One *9*, e113539.

19. Joseph, T.A., Chlenski, P., Litman, A., Korem, T., and Pe'er, I. (2022). Accurate and robust inference of microbial growth dynamics from metagenomic sequencing reveals personalized growth rates. Genome Res. *32*, 558–568.

20. Shao, Y., Forster, S.C., Tsaliki, E., Vervier, K., Strang, A., Simpson, N., Kumar, N., Stares, M.D., Rodger, A., Brocklehurst, P., et al. (2019). Stunted microbiota and opportunistic pathogen colonization in caesarean-section birth. Nature *574*, 117–121.

21. Murray, C.J.L., Ikuta, K.S., Sharara, F., Swetschinski, L., Robles Aguilar, G., Gray, A., Han, C., Bisignano, C., Rao, P., Wool, E., et al. (2022). Global burden of bacterial antimicrobial resistance in 2019: a systematic analysis. Lancet *399*, 629–655.

22. Burnham, P.M., and Hendrixson, D.R. (2018). Campylobacter jejuni: collective components promoting a successful enteric lifestyle. Nat. Rev. Microbiol. *16*, 551–565.

23. Brown, C.L., Mullet, J., Hindi, F., Stoll, J.E., Gupta, S., Choi, M., Keenum, I., Vikesland, P., Pruden, A., and Zhang, L. (2022). mobileOG-db: a manually curated database of protein families mediating the life cycle of bacterial mobile genetic elements. Appl. Environ. Microbiol. *88*, e0099122.

24. Bargheet, A., Noordzij, H.T., Ponsero, A.J., Jian, C., Korpela, K., Valles-Colomer, M., Debelius, J., Kurilshikov, A., and Pettersen, V.K. (2025). Dynamics of gut resistome and mobilome in early life: a meta-analysis. EBioMedicine *114*, 105630.

25. Bargheet, A., Klingenberg, C., Esaiassen, E., Hjerde, E., Cavanagh, J.P., Bengtsson-Palme, J., and Pettersen, V.K. (2023). Development of early life gut resistome and mobilome across gestational ages and microbiota-modifying treatments. EBioMedicine *92*, 104613.

26. Beghini, F., McIver, L.J., Blanco-Míguez, A., Dubois, L., Asnicar, F., Maharjan, S., Mailyan, A., Manghi, P., Scholz, M., Thomas, A.M., et al. (2021). Integrating taxonomic, functional, and strain-level profiling of diverse microbial communities with bioBakery 3. eLife *10*, e65088.

27. Devika, N.T., and Raman, K. (2019). Deciphering the metabolic capabilities of Bifidobacteria using genome-scale metabolic models. Sci. Rep. *9*, 18222.

28. Culp, E.J., and Goodman, A.L. (2023). Cross-feeding in the gut microbiome: Ecology and mechanisms. Cell Host Microbe *31*, 485–499.

29. Reichardt, N., Duncan, S.H., Young, P., Belenguer, A., McWilliam Leitch, C., Scott, K.P., Flint, H.J., and Louis, P. (2014). Phylogenetic distribution of three pathways for propionate production within the human gut microbiota. ISME J. *8*, 1323–1335.

30. Litvak, Y., Mon, K.K.Z., Nguyen, H., Chanthavixay, G., Liou, M., Velazquez, E.M., Kutter, L., Alcantara, M.A., Byndloss, M.X., Tiffany, C.R., et al. (2019). Commensal Enterobacteriaceae protect against Salmonella colonization through oxygen competition. Cell Host Microbe *25*, 128–139.e5.

31. Abdelhamid, A.G., Esaam, A., and Hazaa, M.M. (2018). Cell free preparations of probiotics exerted antibacterial and antibiofilm activities against multidrug resistant E. coli. Saudi Pharm. J. *26*, 603–607.

32. Raheem, A., Liang, L., Zhang, G., and Cui, S. (2021). Modulatory effects of probiotics during pathogenic infections with emphasis on immune regulation. Front. Immunol. *12*, 616713.

33. Xu, J., Duar, R.M., Quah, B., Gong, M., Tin, F., Chan, P., Sim, C.K., Tan, K.H., Chong, Y.S., Gluckman, P.D., et al. (2024). Delayed colonization of Bifidobacterium spp. and low prevalence of B. infantis among infants of Asian ancestry born in Singapore: insights from the GUSTO cohort study. Front. Pediatr. *12*, 1421051.

34. Shao, Y., Wang, S., Gichuki, B.M., Stares, M.D., Rozday, T.J., Kumar, N., Browne, H.P., Dawson, N.J.R., Njunge, J.M., Tigoi, C., et al. (2025). Genomic Atlas of Early Life Bifidobacteria for Rational Development of Next-Generation Probiotics for Infant Health. Cell *S0092–8674*, 54.

35. Gibson, M.K., Wang, B., Ahmadi, S., Burnham, C.A.D., Tarr, P.I., Warner, B.B., and Dantas, G. (2016). Developmental dynamics of the preterm infant gut microbiota and antibiotic resistome. Nat. Microbiol. *1*, 16024.

36. Yassour, M., Vatanen, T., Siljander, H., Hämäläinen, A.M., Härkönen, T., Ryhänen, S.J., Franzosa, E.A., Vlamakis, H., Huttenhower, C., Gevers, D., et al. (2016). Natural history of the infant gut microbiome and impact of antibiotic treatment on bacterial strain diversity and stability. Sci. Transl. Med. *8*, 343ra81–343ra381.

37. Montassier, E., Valdés-Mas, R., Batard, E., Zmora, N., Dori-Bachash, M., Suez, J., and Elinav, E. (2021). Probiotics impact the antibiotic resistance gene reservoir along the human GI tract in a person-specific and antibiotic-dependent manner. Nat. Microbiol. *6*, 1043–1054.

38. Kiu, R., Darby, E.M., Alcon-Giner, C., Acuna-Gonzalez, A., Camargo, A., Lamberte, L.E., Phillips, S., Sim, K., Shaw, A.G., Clarke, P., et al. (2025). Impact of early life antibiotic and probiotic treatment on gut microbiome and resistome of very-low-birth-weight preterm infants. Nat. Commun. *16*, 7569.

39. Pärnänen, K.M.M., Hultman, J., Markkanen, M., Satokari, R., Rautava, S., Lamendella, R., Wright, J., McLimans, C.J., Kelleher, S.L., and Virta, M.P. (2022). Early-life formula feeding is associated with infant gut microbiota alterations and an increased antibiotic resistance load. Am. J. Clin. Nutr. *115*, 407–421.

40. Lebeaux, R.M., Coker, M.O., Dade, E.F., Palys, T.J., Morrison, H.G., Ross, B.D., Baker, E.R., Karagas, M.R., Madan, J.C., and Hoen, A.G. (2021). The infant gut resistome is associated with E. coli and early-life exposures. BMC Microbiol. *21*, 201.

41. Li, X., Stokholm, J., Brejnrod, A., Vestergaard, G.A., Russel, J., Trivedi, U., Thorsen, J., Gupta, S., Hjelmsø, M.H., Shah, S.A., et al. (2021). The infant gut resistome associates with E. coli, environmental exposures, gut microbiome maturity, and asthma-associated bacterial composition. Cell Host Microbe *29*, 975–987.e4.

42. Alcon-Giner, C., Dalby, M.J., Caim, S., Ketskemety, J., Shaw, A., Sim, K., Lawson, M.A.E., Kiu, R., Leclaire, C., Chalklen, L., et al. (2020). Microbiota Supplementation with Bifidobacterium and Lactobacillus Modifies the Preterm Infant Gut Microbiota and Metabolome. Cell Rep. Med. *1*, 100077.

43. Tsukuda, N., Yahagi, K., Hara, T., Watanabe, Y., Matsumoto, H., Mori, H., Higashi, K., Tsuji, H., Matsumoto, S., Kurokawa, K., et al. (2021). Key bacterial taxa and metabolic pathways affecting gut short-chain fatty acid profiles in early life. ISME J. *15*, 2574–2590.

44. Sorbara, M.T., Dubin, K., Littmann, E.R., Moody, T.U., Fontana, E., Seok, R., Leiner, I.M., Taur, Y., Peled, J.U., van den Brink, M.R.M., et al. (2019).

Inhibiting antibiotic-resistant Enterobacteriaceae by microbiota-mediated intracellular acidification. J. Exp. Med. *216*, 84–98.

45. Chang, K.C., Nagarajan, N., and Gan, Y.H. (2024). Short-chain fatty acids of various lengths differentially inhibit Klebsiella pneumoniae and Enterobacteriaceae species. mSphere *9*, e0078123.

46. Pobeguts, O.V., Ladygina, V.G., Evsyutina, D.V., Eremeev, A.V., Zubov, A.I., Matyushkina, D.S., Scherbakov, P.L., Rakitina, D.V., and Fisunov, G.Y. (2020). Propionate Induces Virulent Properties of Crohn's Disease-Associated Escherichia coli. Front. Microbiol. *11*, 1460.

47. World, Health & Organization. WHO Recommendations for Care of the Preterm or Low-Birth-Weight Infant. WHO (2022).

48. Meyer, F., Fritz, A., Deng, Z.L., Koslicki, D., Lesker, T.R., Gurevich, A., Robertson, G., Alser, M., Antipov, D., Beghini, F., et al. (2022). Critical Assessment of Metagenome Interpretation: the second round of challenges. Nat. Methods *19*, 429–440.

49. Langmead, B., and Salzberg, S.L. (2012). Fast gapped-read alignment with Bowtie 2. Nat. Methods *9*, 357–359.

50. Li, H., Handsaker, B., Wysoker, A., Fennell, T., Ruan, J., Homer, N., Marth, G., Abecasis, G., and Durbin, R.; 1000 Genome Project Data Processing Subgroup (2009). The sequence alignment/map format and SAMtools. Bioinformatics *25*, 2078–2079.

51. Blanco-Míguez, A., Beghini, F., Cumbo, F., McIver, L.J., Thompson, K.N., Zolfo, M., Manghi, P., Dubois, L., Huang, K.D., Thomas, A.M., et al. (2023). Extending and improving metagenomic taxonomic profiling with uncharacterized species using MetaPhlAn 4. Nat. Biotechnol. *41*, 1633–1644.

52. Kaminski, J., Gibson, M.K., Franzosa, E.A., Segata, N., Dantas, G., and Huttenhower, C. (2015). High-specificity targeted functional profiling in microbial communities with ShortBRED. PLoS Comput. Biol. *11*, e1004557.

53. van Dijk, L.R., Walker, B.J., Straub, T.J., Worby, C.J., Grote, A., Schreiber, H.L., 4th, Anyansi, C., Pickering, A.J., Hultgren, S.J., Manson, A.L., et al. (2022). StrainGE: a toolkit to track and characterize low-abundance strains in complex microbial communities. Genome Biol. *23*, 74.

54. Li, D., Liu, C.-M., Luo, R., Sadakane, K., and Lam, T.-W. (2015). MEGAHIT: an ultra-fast single-node solution for large and complex metagenomics assembly via succinct de Bruijn graph. Bioinformatics *31*, 1674–1676.

55. Hyatt, D., Chen, G.L., Locascio, P.F., Land, M.L., Larimer, F.W., and Hauser, L.J. (2010). Prodigal: prokaryotic gene recognition and translation initiation site identification. BMC Bioinf. *11*, 119.

56. Buchfink, B., Xie, C., and Huson, D.H. (2015). Fast and sensitive protein alignment using DIAMOND. Nat. Methods *12*, 59–60.

57. Gurevich, A., Saveliev, V., Vyahhi, N., and Tesler, G. (2013). QUAST: quality assessment tool for genome assemblies. Bioinformatics *29*, 1072–1075.

58. Schmid, R., Heuckeroth, S., Korf, A., Smirnov, A., Myers, O., Dyrlund, T.S., Bushuiev, R., Murray, K.J., Hoffmann, N., Lu, M., et al. (2023). Integrative analysis of multimodal mass spectrometry data in MZmine 3. Nat. Biotechnol. *41*, 447–449.

59. Nothias, L.-F., Petras, D., Schmid, R., Dührkop, K., Rainer, J., Sarvepalli, A., Protsyuk, I., Ernst, M., Tsugawa, H., Fleischauer, M., et al. (2020). Feature-based molecular networking in the GNPS analysis environment. Nat. Methods *17*, 905–908.

60. Dührkop, K., Fleischauer, M., Ludwig, M., Aksenov, A.A., Melnik, A.V., Meusel, M., Dorrestein, P.C., Rousu, J., and Böcker, S. (2019). SIRIUS 4: a rapid tool for turning tandem mass spectra into metabolite structure information. Nat. Methods *16*, 299–302.

61. Shannon, P., Markiel, A., Ozier, O., Baliga, N.S., Wang, J.T., Ramage, D., Amin, N., Schwikowski, B., and Ideker, T. (2003). Cytoscape: A Software Environment for Integrated Models of Biomolecular Interaction Networks. Genome Res. *13*, 2498–2504.

62. Pang, Z., Lu, Y., Zhou, G., Hui, F., Xu, L., Viau, C., Spigelman, A.F., MacDonald, P.E., Wishart, D.S., Li, S., et al. (2024). MetaboAnalyst 6.0: towards a unified platform for metabolomics data processing, analysis and interpretation. Nucleic Acids Res. *52*, W398–W406.

63. Kuznetsova, A., Brockhoff, P.B., and Christensen, R.H.B. (2017). lmerTest Package: Tests in Linear Mixed Effects Models. J. Stat. Softw. *82*, 1–26.

64. Lin, H., and Peddada, S.D. (2020). Analysis of compositions of microbiomes with bias correction. Nat. Commun. *11*, 3514.

65. Wickham, H. (2016). ggplot2: Elegant Graphics for Data Analysis (Springer).

66. Gu, Z., and Hübschmann, D. (2022). Make interactive complex heatmaps in R. Bioinformatics *38*, 1460–1462.

67. Rohart, F., Gautier, B., Singh, A., Lê Cao, K.A., and mixOmics, K.-A. (2017). An R package for 'omics feature selection and multiple data integration. PLoS Comput. Biol. *13*, e1005752.

68. Langfelder, P., and Horvath, S. (2008). WGCNA: an R package for weighted correlation network analysis. BMC Bioinf. *9*, 559.

69. Bargheet, A. (2025). AssumpSure: a user-friendly R Shiny package for automated validation of statistical assumptions and appropriate test selection. J. Open Source Softw. *10*, 9286.

70. Kuwelker, K., Langeland, N., Löhr, I.H., Gidion, J., Manyahi, J., Moyo, S.J., Blomberg, B., and Klingenberg, C. (2021). Use of probiotics to reduce infections and death and prevent colonization with extended-spectrum beta-lactamase (ESBL)-producing bacteria among newborn infants in Tanzania (ProRIDE Trial): study protocol for a randomized controlled clinical trial. Trials *22*, 312.

71. Jia, B., Raphenya, A.R., Alcock, B., Waglechner, N., Guo, P., Tsang, K.K., Lago, B.A., Dave, B.M., Pereira, S., Sharma, A.N., et al. (2017). CARD 2017: expansion and model-centric curation of the comprehensive antibiotic resistance database. Nucleic Acids Res. *45*, D566–D573.

72. Diebold, P.J., Rhee, M.W., Shi, Q., Trung, N.V., Umrani, F., Ahmed, S., Kulkarni, V., Deshpande, P., Alexander, M., Thi Hoa, N., et al. (2023). Clinically relevant antibiotic resistance genes are linked to a limited set of taxa within gut microbiome worldwide. Nat. Commun. *14*, 7366.

73. Wick, R.R., Howden, B.P., and Stinear, T.P. (2025). Autocycler: long-read consensus assembly for bacterial genomes. Bioinformatics *41*, btaf474.

74. Han, J., Lin, K., Sequeira, C., and Borchers, C.H. (2015). An isotope-labeled chemical derivatization method for the quantitation of short-chain fatty acids in human feces by liquid chromatography–tandem mass spectrometry. Anal. Chim. Acta *854*, 86–94.

75. Grijseels, S., Vasskog, T., Heinsvig, P.J., Myhre, T.N., Hansen, T., and Mardal, M. (2024). Validation of two LCHRMS methods for large-scale untargeted metabolomics of serum samples: Strategy to establish method fitness-for-purpose. J. Chromatogr. A *1732*, 465230.

76. Dührkop, K., Shen, H., Meusel, M., Rousu, J., and Böcker, S. (2015). Searching molecular structure databases with tandem mass spectra using CSI:FingerID. Proc. Natl. Acad. Sci. USA *112*, 12580–12585.

77. Djoumbou Feunang, Y., Eisner, R., Knox, C., Chepelev, L., Hastings, J., Owen, G., Fahy, E., Steinbeck, C., Subramanian, S., Bolton, E., et al. (2016). ClassyFire: automated chemical classification with a comprehensive, computable taxonomy. J. Cheminform. *8*, 61.

78. Rao, C., Coyte, K.Z., Bainter, W., Geha, R.S., Martin, C.R., and Rakoff-Nahoum, S. (2021). Multi-kingdom ecological drivers of microbiota assembly in preterm infants. Nature *591*, 633–638.

STAR★METHODS

KEY RESOURCES TABLE

REAGENT or RESOURCE	SOURCE	IDENTIFIER
**Biological samples**		
fecal samples	This study	NA
**Bacterial and virus strains**		
L. acidophilus NCFM, *B. bifidum* Bb-06, *B. longum* subsp. *infantis* Bi-26 (Labinic® drops)	Biofloratech Ltd, UK	NA
**Chemicals, peptides, and recombinant proteins**		
eSwab® collection system	Copan Diagnostics, USA	Cat# 490CE.A
OMNIgene·GUT DNA kit	DNA Genotek, Canada	OM-200
OMNImet·GUT metabolite kit	DNA Genotek, Canada	ME-200
ChromID ESBL agar	bioMérieux, France	Cat# 43861
Analytical and internal standards (SCFAs, OAs, 13C-3NPH)	Sigma-Aldrich/Cayman Chemicals	Various
**Other**		
Waters XEVO TQ-XS Triple Quadrupole MS	Waters Corporation	NA
Thermo Orbitrap ID-X Tribrid MS	Thermo Fisher Scientific	NA
**Deposited data**		
Shotgun metagenomic reads	NCBI SRA	PRJNA1305341
ESBL-E isolate genomes	NCBI SRA	PRJNA1370689
Untargeted metabolomics dataset	MassIVE	MSV000098821
MobileOG marker families	Zenodo	https://doi.org/10.5281/zenodo.11491353
Custom analysis scripts	Zenodo	https://doi.org/10.5281/zenodo.15626017
**Software and algorithms**		
FastQC (v.11.9)	N/A	https://qubeshub.org/resources/fastqc/about
Trim Galore (v.0.6.10)	N/A	https://www.bioinformatics.babraham.ac.uk/projects/trim_galore/
Bowtie 2 (v.2.4.4)	Langmead, B. & Salzberg, S.L.[49]	https://doi.org/10.1038/nmeth.1923
SAMtools (v.1.12)	Li, H. et al.[50]	https://doi.org/10.1093/bioinformatics/btp352
MetaPhlAn4 (v.4.0.6)	Blanco-Míguez, A. et al.[51]	https://doi.org/10.1038/s41587-023-01688-w
CoPTR (v.1.1.4)	Joseph, T.A. et al.[19]	https://doi.org/10.1101/gr.275533.121
ShortBRED (v.0.9.5)	Kaminski, J. et al.[52]	https://doi.org/10.1371/journal.pcbi.1004557
MobileOG (v.1.6)	Brown, C.L. et al.[23]	https://doi.org/10.1128/aem.00991-22
StrainGE (v.1.3.3)	Van Dijk, L.R. et al.[53]	https://doi.org/10.1186/s13059-022-02630-0
HUMAnN (v.3.0.1)	Beghini, F. et al.[26]	https://huttenhower.sph.harvard.edu/humann/
MEGAHIT (v.1.2.9)	Li, D. et al.[54]	https://doi.org/10.1093/bioinformatics/btv033
ABRicate (v.1.0.1)	N/A	https://github.com/tseemann/abricate
Prodigal (v.2.6.3)	Hyatt, D. et al.[55]	https://doi.org/10.1186/1471-2105-11-119
DIAMOND (v. 2.1.6)	Buchfink, B. et al.[56]	https://doi.org/10.1038/nmeth.3176
Dorado (v.7.6.7)	N/A	https://github.com/nanoporetech/dorado
Filtlong (v.0.2.1)	N/A	https://github.com/rrwick/Filtlong
Autocycler (v.0.4.0)	N/A	https://github.com/rrwick/Autocycler
QUAST (v.5.2.0)	Gurevich, A. et al.[57]	https://github.com/ablab/quast
Speciator (v.4.0.0)	N/A	https://github.com/pathogenwatch-oss/speciator
MZmine (4.7.8)	Schmid, R. et al.[58]	https://mzmine.github.io
GNPS	Nothias, L.-F. et al.[59]	https://gnps.ucsd.edu
SIRIUS (v.6.2.2)	Dührkop, K. et al.[60]	https://bio.informatik.uni-jena.de/software/sirius

(*Continued on next page*)

Continued

REAGENT or RESOURCE	SOURCE	IDENTIFIER
Cytoscape (v.3.10.3)	Shannon, P. et al.[61]	https://cytoscape.org
MetaboAnalyst (v.6.0)	Pang, Z. et al.[62]	https://www.metaboanalyst.ca
R (v.4.3.1)	R Core Team	https://www.r-project.org/
vegan (v.2.5.7)	N/A	https://cran.r-project.org/web/packages/vegan/vegan.pdf
lmerTest (v.3.1.3)	Kuznetsova, A. et al.[63]	https://cran.r-project.org/web/packages/lmerTest/lmerTest.pdf
emmeans (v.1.10.7)	N/A	https://cran.r-project.org/package=emmeans
ANCOM-BC (v.2.2.2)	Lin, H. & Peddada, S.D.[64]	https://bioconductor.org/packages/release/bioc/html/ANCOMBC.html
MaAsLin 3 (v.0.99.4)	Nickols WA et al.[17]	https://github.com/biobakery/Maaslin3
randomForest (v.4.7.1.2)	N/A	https://cran.r-project.org/package=randomForest
caret (v.6.0.94)	N/A	https://cran.r-project.org/package=caret
MASS (v.7.3.65)	N/A	https://cran.r-project.org/package=MASS
Hmisc (v.5.1.3)	N/A	https://cran.r-project.org/package=Hmisc
ggplot2 (v.3.3.6)	Wickham, H.[65]	https://cran.r-project.org/web/packages/ggplot2/ggplot2.pdf
ComplexHeatmap (v.2.20.0)	Gu, Z. & Hübschmann, D.[66]	https://bioconductor.org/packages/ComplexHeatmap
plspm (v.0.5.1)	N/A	https://cran.r-project.org/package=plspm
mixOmics (v.6.28.0)	Rohart, F. et al.[67]	https://mixomics.org
WGCNA (v.1.73)	Langfelder, P. & Horvath, S.[68]	https://cran.r-project.org/web/packages/WGCNA/index.html
AssumpSure (v1.0.0)	Bargheet, A.[69]	https://cran.r-project.org/web/packages/AssumpSure/index.html
R code	N/A	https://doi.org/10.5281/zenodo.18644563

EXPERIMENTAL MODEL AND STUDY PARTICIPANT DETAILS

Ethics statement

Ethical approvals for the ProRIDE trial were obtained from the Regional Committee for Medical and Health Research Ethics in Western Norway (REK Vest 2019/1025), the Tanzanian National Institute of Medical Research (NIMR/HQ/R.8a/Vol.IX/3398), and the Tanzania Medicines and Medical Devices Authority (Authorization no.TZ21CT0002).

Study design and participants

The ProRIDE trial was an investigator-initiated, single-site, double-blind, placebo-controlled RCT (1:1 ratio) conducted at Haydom Lutheran Hospital (HLH) and the surrounding area in North-East Tanzania.[15,70] A total of 2,000 healthy infants with a birth weight of $\geq$2.0 kg were enrolled within the first three days of life and randomly assigned to receive either a multistrain probiotic (n = 1,000) or a placebo (n = 1,000). Caregivers were instructed to administer 5 drops once daily from enrollment until the bottle was empty, corresponding to about 4 weeks of age. This study focuses on a subset of 152 participants (Table S1) for whom deep metagenomic sequencing of fecal samples was performed at 6 weeks and 6 months of age (250 samples). Additionally, samples from this subset were analyzed using both untargeted (200 samples from 101 infants) and targeted (241 samples from 152 infants) metabolomics profiling.

The intervention group received Labinic probiotic drops (Bifloratech Ltd, Surrey, UK). Each daily dose of five drops (0.2 mL) contained 2 billion colony-forming units (CFU) of *Lactobacillus acidophilus* NCFM, *Bifidobacterium bifidum* Bb-06, and *Bifidobacterium longum* subsp. *infantis* Bi-26, with equal amounts of all three strains. Each batch of Labinic undergoes manufacturer-performed GMP quality control, including verification of viable CFU counts for all probiotic strains. Independent CFU quantification was not performed as part of this study. The placebo consisted of inactive ingredients. Both solutions were identical in taste, color, and packaging. Trained field workers conducted home visits at 1 week, 6 weeks, and 6 months of life. During these visits, a standardized electronic case report form was administered to collect data on recent illnesses, medication use, breastfeeding practices, growth, and the child's overall condition. The field workers also collected a stool sample at both the 6-week and 6-month visits. The sample was transferred to eSwab (Copan Diagnostics, CA, USA) and two collection tubes from DNA Genotek (Ottawa, Canada), which stabilize

fecal DNA (OMNIgene·GUT | OM-200) and metabolites (OMNImet·GUT | ME-200). It was then transported in cold bags to HLH and stored at −80°C. The eSwab and the OM/ME-200 tubes were later transported on dry ice to Norway.[15]

The metadata were collected from clinical records and caregiver interviews. For this sub-study specifically, the data included sex (male or female), hospitalization (defined as any inpatient admission, excluding the immediate post-delivery stay unless extended for medical reasons), place of birth (categorized as HLH, other health facility and home delivery), antibiotic exposure (assessed during follow-up visits at 6 weeks and 6 months post-enrollment using structured case report forms), and detection of ESBL-E carriage by culture.

METHOD DETAILS

DNA extraction from human stool samples and sequencing

Fecal samples were thawed and mixed with liquefaction reagent (DNA Genotek, Canada). To each sample, 10 μL of a *Vibrio campbellii* culture (10^4 cells/μL) was added as an internal spike-in control. A total of 800 μL of the mixture was transferred to ZR BashingBead lysis tubes containing a combination of 0.1- and 0.5-mm beads (Zymo Research, USA) for mechanical lysis. Each extraction batch included a positive and a negative control: the positive control consisted of 75 μL of the ZymoBIOMICS Microbial Community Standard (Zymo Research, USA) in 725 μL liquefaction reagent, while the negative control consisted of 700 μL liquefaction reagent and 100 μL of *Vibrio campbellii* culture (10^4 cells/μL).

Samples were mechanically lysed using the FastPrep-24 5G instrument (MP Biomedicals, USA) and subsequently centrifuged at 10,000 × g for 1 min. A volume of 200 μL of the resulting supernatant was transferred to a deep-well plate for DNA extraction. The extraction was performed using the Tecan Fluent liquid-handling platform (Tecan, Switzerland) according to the ZymoBIOMICS 96 MagBead DNA protocol (Zymo Research). DNA was eluted in 50 μL of EB buffer (Qiagen, Germany) and stored at −20°C.

DNA concentrations were quantified using the QuantIT High Sensitivity dsDNA Assay on a Tecan Spark plate reader (Tecan, Switzerland). Samples with DNA concentrations above 1.1 ng/μL were spiked with 1% *Alicyclobacillus acidophilus* DNA (relative to total input), while samples with concentrations below 1.1 ng/μL were supplemented with *Alicyclobacillus* DNA to reach a total of 50 ng per reaction. The negative sequencing control was also spiked with *Alicyclobacillus* DNA to monitor potential background signal.

Library preparation was performed using 50 ng of input DNA and the MGI FS Library Prep Set (MGI Tech, China). Library quality was assessed using the Agilent TapeStation D1000 kit (Agilent Technologies, USA), and concentrations were confirmed using the same QuantIT assay. Equimolar amounts of the resulting libraries were pooled to a final concentration of 100 pM, circularized using the MGI Easy Circularization Kit (MGI Tech, China), and sequenced on the DNBSEQ-T7 platform (MGI Tech, China) using 150 bp paired-end reads.

ESBL-E screening

Fecal samples collected with eSwab were cultured using chromogenic agars selective for ESBL-E (ChromID ESBL, BioMérieux, Marcy l'Étoile, France). All samples were also plated on non-selective blood agar for growth control. Agar plates were incubated at 35°C under normal conditions for 18–24 h before inspection. When no growth was observed on blood agar, the sample was considered invalid. Samples that showed no growth on the chromogenic agars after 18–24 h of incubation were reported as negative for ESBL-E. ESBL-E suspect colonies were identified at the species level using MALDI-TOF MS (Bruker Daltonik). An ESBL-E phenotype was confirmed using the double disk approximation test (Liofilchem, Roseto degli Abruzzi, Italy).

Bioinformatics preprocessing of metagenomic data

Pair-end reads were checked for quality using FastQC v.0.11.9. The adaptor sequences and low-quality reads were filtered using Trim Galore v.0.6.10 with the default parameters. The human DNA contaminant sequences were discarded from all samples by filtering out reads mapped to the human reference genome (GRCh38, downloaded from NCBI GenBank in 2022) using Bowtie2 v.2.4.43 with –very-sensitive *–end-to-end* parameters. The identified paired reads that did not map against the human genome using SAMtools v.1.12[50] with *-f 12 -F 256* were used in subsequent analyses.

Metagenome, resistome, and mobilome profiling

Taxonomic profiling was obtained using MetaPhlAn4 v.4.0.6[51] against the CHOCOphLAN database. The resistome annotation of metagenomic reads was performed by mapping them against the *nucleotide_fasta_protein_homolog_model* from the Comprehensive Antibiotic Resistance Database (CARD[71] v.3.2.9) database using Bowtie2 v.2.4.4[49] with parameter *–very-sensitive-local*. For ARG annotation, a coverage threshold of 80% was used. Using SAMtools v.1.12,[50] the mapped reads were separated from the unmapped reads, sorted, and indexed, and the number of reads mapped for each ARG was calculated. The counts were then normalized for each sample to the total gene length by calculating reads per kilobase reference per million mapped reads (RPKM). The clinical relevance of individual ARGs was based on the AMR Gene Family previously reported as clinically relevant by Diebold et al.[72] and the National Database of Antibiotic Resistant Organisms (NDARO). Specifically, we excluded ARGs encoding efflux pumps and other metabolic functions because distinguishing their physiological roles from antibiotic resistance is difficult.

Although this filtering enriches the dataset for clinically relevant, horizontally acquired ARGs, metagenomic annotation cannot always fully distinguish intrinsic from acquired resistance determinants; therefore, some degree of overlap may remain.

The relative abundance of MGEs was quantified by employing ShortBRED v.0.9.5.[52] Specifically, MGEs sourced from the MobileOG v.1.6[23] served as the proteins of interest for identifying marker families using '*shortbred_identify.py*' with *'–clustid 0.95'* option. MGEs' read counts were normalized in RPKM using '*shortbred_quantify.py*'. We also employed a computed peak-to-trough ratio (CoPTR) v.1.1.4[19] to estimate the impact of probiotic supplementation on the replication rates of the two most important ESBL-E bacteria, *E. coli* and *K. pneumoniae*. Strain analysis was performed using StrainGST from StrainGE v.1.3.3.[53] Reference genomes for *B. longum* were downloaded from NCBI on 20 October 2025 (n = 102 genomes). The database was built with StrainGE and is available on Zenodo (https://doi.org/10.5281/zenodo.17671286). The quality-filtered short-read sequences were assembled into longer contiguous sequences (contigs) using MEGAHIT v.1.2.9[54] with the default parameters. For assembly assessment, MetaQUAST from QUAST v.5.2.0[57] was used with the *-m* 1000 option (Table S16). Functional pathway profiling was performed using HUMAnN v3.0.1,[26] generating both community-level and species-resolved MetaCyc pathway abundances from the quality-filtered metagenomic reads. The per-sample pathway abundance tables were merged with *humann_join_tables* and normalised to relative abundances using *humann_renorm_table* for downstream analyses. Pathway tables were normalized and analyzed for differential abundance using the Mann–Whitney U test with Benjamini–Hochberg FDR correction. Analyses were conducted separately for unstratified (community-level) and stratified (species-level) outputs, focusing on the High and Low *Bifidobacterium* clusters.

Linking ARGs in metagenomes to ESBL-E isolates

To validate the hosts of the detected ARGs, we linked ESBL-E isolate genomes to their corresponding metagenomic samples. Among the 250 samples, 47 had a corresponding cultured ESBL-E isolate. Low-quality or unsequenced isolates were removed (n = 10), and to ensure unambiguous metagenome-isolate linkage, samples with more than one recovered ESBL-E isolate were excluded (n = 7). The resulting 32 samples were whole-genome sequenced on the Oxford Nanopore Technologies (ONT) GridION platform using R10.4.1 flow cells and the rapid SQK-RBK114-96 barcoding kit (ONT, Oxford, UK). DNA was extracted using the GenFind V3 reagent kit with the Bacteria protocol (Beckman Coulter Life Sciences, Indianapolis, USA). Base calling was performed with Dorado v7.6.7 using the super accurate model (dna_r10.4.1_e8.2_400bps_sup@v4.3.0). The reads were filtered with filtlong v0.2.1 (https://github.com/rrwick/Filtlong), and genome assemblies generated with Autocycler[73] v0.4.0 (https://github.com/rrwick/Autocycler), and all genomes were fully circularized. The quality was assessed using fast_count (https://github.com/rrwick/MinION-desktop) and quast[57] v5.2.0 (https://github.com/ablab/quast). The genome sequences have been deposited under BioProject PRJNA1370689; the accessions are listed in Table S12. ARGs in both the isolate and metagenome assemblies were identified using Abricate v1.0.1 (https://github.com/tseemann/abricate) with the CARD database v4.0.1, applying a 99% sequence identity and length threshold. ESBL-encoding genes were assigned based on annotation in CARD_AMR_clustered.csv (https://github.com/klebgenomics/Kleborate; version 2025-05-13). Species were identified with Speciator v4.0.0 (https://github.com/pathogenwatch-oss/speciator).

Analysis of ESBL genes in isolates and corresponding metagenomes

The 32 ESBL-E isolates and 33 ESBL genes were selected for resistome comparison. Abricate v1.0.1 with CARD database v4.0.1 was used with a 99.0% sequence identity and length threshold to identify ARGs for the isolate-metagenome comparison. To identify the taxonomy, open Reading Frames (ORFs) were predicted from contigs exceeding 1000 base pairs that harbored ARGs identified by ABRicate against CARD, using Prodigal v.2.6.3[55] with default parameters. Following this, we determined the taxonomy by comparing amino acid sequences from Prodigal against the NCBI non-redundant database, executed using DIAMOND v. 2.1.6[56] with the following options: *'–evalue 0.00001', '–id 95', and '–query-cover 95'*. For contigs assigned to multiple species, we retained only those where at least 75% of ORFs were assigned to a single species.

Mass spectrometry (MS)-based metabolomics analyses

For both targeted and untargeted analyses, aliquots of fecal samples collected in OMNImet·GUT were vortexed for 1 min and centrifuged at 6,500 × g for 10 min at 4°C. The supernatants were spin-filtered using a 10 kDa cutoff spin filter at 20,800 × g for 10 min at 0°C. An aliquot of non-processed fecal sample (≈100 μL) was dried to determine the wet-to-dry weight ratio. This ratio was used to estimate theoretical dry weight of the samples analyzed. The metabolite levels were further adjusted for dilution and extraction volume. Final peak areas were normalized by dividing the final signal area by the estimated dry weight, yielding peak areas per gram of dried feces.

UPLC-MS/MS analysis for quantification of short-chain fatty acids and organic acids

For targeted profiling of short-chain fatty acids (SCFAs) and organic acids (OAs), samples were prepared according to a previously validated method[74] with minor modifications. In brief, cleaned supernatants were derivatized in a 2:1:1 volume ratio with 200 mM 3-nitrophenylhydrazine (3NPH) and a 120 mM N-(3-dimethylaminopropyl)-N′-ethylcarbodiimide (EDC) solution (6% pyridine) prepared in a 50:50 (v/v) acetonitrile (ACN): MilliQ water mixture. The derivatization reaction was allowed to proceed for 30 min at 40°C. The reaction mixture was first diluted 1:2 with 2,6-di-*tert*-butyl-4-methylphenol (BHT) and subsequently with 2% formic acid, both prepared in ACN:MilliQ (10:90, v/v), to quench the reaction. Lastly, the quenched mixture was diluted at 1:6.25 ratio

with ACN: MilliQ (10:90, v/v), yielding a total dilution after derivatization of 1:25. Before MS analysis, the extract was mixed with an equal volume of isotopically labeled internal standards (ISTD_T).

Preparation of external and internal standards and quality controls

Analytical standards for 15 SCFAs and 8 OAs were purchased from Sigma-Aldrich (Darmstadt, Germany): acetate, propionate, butyrate, isobutyrate, valerate, isovalerate, pivalate, 2-methylbutyrate, methyl isobutyrate, hexanoate, 3,3-dimethylbutyrate, 2-ethylbutyrate, 2-methylvalerate, 3-methylvalerate, 4-methylvalerate, lactate, succinate, pyruvate, oxaloacetate, α-ketoglutarate, fumarate, malate, and citrate. Analytical grade 3NPH, EDC, pyridine, formic acid, and BHT were also purchased from Sigma-Aldrich. For ISTD_T preparation, ^{13}C-3NPH was purchased from Cayman Chemicals.

Preparation of external standard (ESTD) curves and ISTD_T for all targeted metabolites was performed as previously described.[74] In brief, 20 mM ESTD mixes of 1) acetate, propionate, and butyrate, 2) the remaining SCFA, and 3) all OAs, were freshly prepared in 50% ACN:MilliQ (v/v). The standard mixes were diluted to 5 mM for acetate, propionate, and butyrate, and one mM for the rest. This mixture was further diluted to form an 8-point calibration curve, with a 2-fold dilution between the two highest concentrations, followed by a 3-fold dilution between the other concentrations (3.43–5000 μM for acetate, propionate, and butyrate, and 0.69–1,000 μM for the rest). Because lactate levels in infant feces were much higher than those of any other metabolite, an additional standard mixture for lactate was prepared at 30 mM and diluted to a 6-point curve spanning 937.5–30,000 μM, with a 2-fold dilution between points. The two sets of standards were then derivatized and processed under the same conditions and at the same concentrations as described for the fecal samples above. A fecal sample from an adult donor was processed in the same manner as the standards/samples to serve as an internal quality control (QC) sample. It was injected every 10 samples throughout the analytical run. ISTD_T mixes were prepared by diluting acetate to 4 mM, propionate and butyrate to 2 mM, and the remaining metabolites to 1 mM. Fifty μL of each ISTD_T was mixed with 1 mg of 13C-3NPH, 25 μL EDC-6% pyridine, and 25 μL 50% ACN: MilliQ (v/v). The mixture was derivatized under the same conditions as above and, after adding BHT and formic acid, diluted to 100 mL with 10% CAN.

Targeted UPLC-MS/MS instrumentation and data processing

Targeted metabolomic analysis was conducted using Waters XEVO TQ-XS Triple Quadrupole mass spectrometer coupled to Waters Acquity UPLC. Chromatographic separation was achieved by gradient elution on Acquity Premier HSS T3 (1.8 μm, 2.1 × 100 mm). Mobile phase A consisted of MilliQ water with 0.1% formic acid (v/v), and mobile phase B of ACN with 0.1% formic acid (v/v). The gradient was as follows: 15% B for 2 min, 15–40% B in 10 min, 40–100% B in 1 min, 100% B for 0.1 min, and held at 15% B for 2 min. The flow rate was 0.5 mL/min, the column temperature 50°C, and the autosampler was maintained at 6°C. The mass spectrometer was operated in negative electrospray ionization mode using multiple reaction monitoring. Desolvation temperature was 550°C, desolvation gas flow 1000 L/h, cone gas flow 150 L/h, nebulizer gas flow 7 bar, capillary voltage 0.6 kV, and ion energy 1 and 2 were set to 1 and 2, respectively. Peak integration and data processing were performed using the TargetLynx application in MassLynx (version 4.2 SCN 1007). Analyte responses were calculated as a ratio of the endogenous peak area to the corresponding ISTD_T peak area. Quantification was based on a weighted ($1/x^2$) linear regression of the ESTD curve, using at least six calibration points that covered the biological concentration range, with an R^2 value close to 0.99. Two metabolite pairs, isovalerate/methylisobutyrate and pivalate/2-methylbutyrate, did not separate and were thus quantified together.

Untargeted LC-MS/MS profiling

Supernatants from fecal samples were prepared as for the targeted analysis. Five μL of each sample supernatant was pooled to generate a QC sample. Afterward, all supernatants including the QC were diluted 1:10 with ACN: MilliQ water (90:10, v/v) containing internal standard mix (ISTD_U) of Acetyl-/-carnitine-D_3, Hexadecanoyl(palmitoyl)-/-carnitine-D_3, l-Leucine-5,5,5-D_3, l-Tryptophan-(indole-D_5), l-Methionine-(methyl-D_3), Stearic acid-18,18,18-D_3, Chenodeoxycholic-2,2,3,4,4,6,6,7,8-D_9 acid, 18:0-D_{35} Lyso PC, Dopamine-1,1,2,2-D_4 hydrochloride (Merck Life Science, Darmstadt, Germany). Before analysis, the dilution factor, solvent type, sample extraction method, and sample stability in the autosampler, freezer, and at room temperature were evaluated with respect to efficient sample handling, peak shape and intensity, and reproducibility, and were found to be well within the instrument's dynamic range. The final samples for analysis were randomized (RAND function in Excel, Microsoft Office), transferred to 96-well injection plates (Waters, SKU: 186002481), with the QC sample included every 10 injections, and stored in the autosampler (6°C) throughout acquisition.

LC-MS/MS instrumentation and data acquisition

Untargeted metabolomic profiling was based on a published hydrophilic interaction liquid chromatography (HILIC) method.[75] In summary, the instrument was a Thermo Vanquish UHPLC system coupled with a Thermo Scientific Orbitrap ID-X Tribrid Mass Spectrometer. The data acquisition was performed using Thermo AcquireX Deep Scan module in data-dependent acquisition (DDA). Chromatographic separation was achieved by injecting 3 μL of each sample through a BEH Amide (100 × 2.1 mm, 1.7 Å) column at a flow rate of 0.45 mL/min. The column temperature was maintained at 50°C, and the autosampler at 6°C. The separation was carried out using gradient elution with mobile phase A consisting of H_2O with 10 mM NH_4Ac at pH 9, and mobile phase B of ACN: H_2O (9:1, v/v) with 10 mM NH_4Ac at pH 9. Mobile phase B was maintained at 100% for 6 min to equilibrate the system and then kept at 100% for an additional 2.5 min after injection. Then 100-60% from 2.5–9 min and kept at 60% for 0.2 min.

CellPress
OPEN ACCESS

The ion source was operated in heated negative electrospray ionization (2500 V). Gas settings were 50 arbitrary units (arb) for sheath gas, 10 arb for auxiliary gas, and one arb for sweep gas, with an ion transfer tube temperature of 325°C and vaporizer temperature of 350°C. Full MS scans were acquired using the Orbitrap at a resolution of 120,000 over a scan range of 70–800 m/z. The automatic gain control (AGC) target was set to custom with a normalized AGC of 25 and a maximum injection time of 50 milliseconds (ms). For MS/MS, up to 8 dependent scans were triggered per MS1 event using higher-energy collision dissociation (HCD, with stepped collision energies of 20%, 35%, and 50%) and collision-induced dissociation (CID, with a fixed energy of 30%). MS2 scans were also acquired in the Orbitrap at a resolution of 30,000 with a quadrupole isolation window of 1.2 m/z and a maximum injection time of 150 ms. Dynamic exclusion was enabled (excluding after one acquisition, for 5 s) with isotope exclusion to prevent repeated fragmentation of isotopologues.

Untargeted LC-MS/MS quality control assessment

Prior to sample injection, a system blank consisting of clean sample solvent (ACN: MilliQ, 90:10, v/v) was injected to ensure that the instrument and mobile phase were free from contamination. Next, several injections of a system control sample consisting of 0.1 mg/L quercitin, amitriptylin, histidine, arginine, labetalol, doxepin, proline, and tryptophan (Merck Life Science, Darmstadt, Germany) were injected to monitor and ensure instrument performance, *e.g.*, retention time, mass accuracy, peak shape, and peak intensity. When all the above quality checks were acceptable, a processed and diluted clean solvent from OMNImet·GUT | ME-200 tubes was injected twice as an extraction blank. Then, four injections of the pooled QC were performed to generate deep-scan ID samples, after which unknown samples were run in full-scan mode. The pooled QC was injected every 10 samples to monitor drift along the run.

Additionally, the ISTD_U spike in every sample was used to monitor drift and injection quality. After the injection sequence, the ISTD_U m/z values were uploaded into the software Skyline (v 24.1.0.414, MacCross Lab, University of Washington, USA), where retention time drift and signal intensity were monitored for each ISTD_U in each sample. Next, extracted ion chromatograms were manually checked and compared in the software Freestyle (Thermo Fisher Scientific, San Jose, USA). Specific masses for tryptophan, proline, and isoleucine were manually verified in each QC to confirm peak shape, retention time, and intensity. The blanks were also checked to verify the absence of the specific masses. Finally, to evaluate stability, QCs were visualized in a PCA plot to detect potential global drift in metabolomic profiles over time.

Raw MS/MS data processing

The raw data files were centroided and processed using MZmine 4.7.8[58] in two processing batches. The processing workflows were as follows: in the first batch, mass detection in MS1 was performed for all samples using a noise level of 1.0e4 based on precursor intensity. Then, mass detection in MS2 was performed without noise-level filtering across four pooled MS2 samples, which were representative of the MS2 data. Subsequent steps included chromatogram builder, smoothing (Savitzky-Golay), local minimum feature resolver, 13C isotope filter, and isotopic peaks finder with default settings. Processing batch two was performed by first running the join aligner on the merged ID samples, then aligning to all the full-scan samples with a retention time tolerance of 0.2 min. The extraction blank was used in the module feature list subtraction, with a fold change threshold of 300%, to retain relevant features. The following modules were applied: group MS2 scans with features, feature list rows filter, correlation grouping, and ion identity networking. The processed data were extracted for downstream analysis in GNPS[59] and SIRIUS.[60] To determine the need for drift correction, the quantification table was uploaded to MetaboAnalyst 6.0, and the QCs were monitored using principal component analysis (PCA).

SIRIUS-based annotation

For feature annotation, the processed data from MZmine were uploaded into SIRIUS 6.2.2[60] using default settings. Spectral matching, molecular formula prediction, CSI: FingerID,[76] and structure database search with PubChem as a fallback was enabled. Annotations were manually curated and evaluated based on the compound's confidence score. A score above 0.6 was considered good if the substructure matched with databases and had a consistent top structure hit. These features were assigned a level 2 annotation. Features with lower confidence scores, which showed reliable substructure matches, were assigned level 3 annotation and were annotated at the ClassyFire[77] most specific class level.

Feature-based molecular networking and statistics

For feature-based molecular networking, the processed data from MZmine were uploaded and run in GNPS2.[59] The processed network was then uploaded to the software Cytoscape 3.10.3[61] for network visualization. Edges were labeled with their delta mz and further colored by cosine (blue) or MS1 annotation (red). Furthermore, the node sizes were mapped to the sum of precursor intensity.

QUANTIFICATION AND STATISTICAL ANALYSIS

Linear mixed models and differential abundance analyses

The α-diversity analysis was performed using *vegan* v.2.5.7 R package. We evaluate the statistical influence of variables on the diversity and relative abundance of the infant microbiota, resistome, and mobilome using linear mixed-effects modeling (LMM) with the

lmerTest v.3.1.3 R package with 'infant_id' as a random effect. To standardize the resistome and mobilome relative abundances, we applied an inverse normal transformation (*qnorm* function in R). Additionally, to detect collinearity among the variables, we used a variance inflation factor ($\leq$5) using the *car* v.3.1.3 R package. We used the *emmeans* v.1.10.7 R package to compute estimated marginal means following LMM to evaluate the effect of covariates at each time point. Linear regression models with the Benjamini-Hochberg procedure for *p*-value adjustment were employed to identify differentially abundant SCFAs, considering 'trial_arm', 'sex', 'ESBL-E', 'born_hospital', and 'antibiotics' as binary covariates (yes/no). The *ANCOM-BC* v.2.2.2[64] R package with the Benjamini-Hochberg procedure for *p*-value adjustment was used to identify differentially abundant features at the species level. In this model, we considered 'trial_arm', 'sex', 'ESBL-E', 'born_hospital', and 'antibiotics' as fixed effects. Differential abundance was considered statistically significant at $p < 0.05$ and $q < 0.25$, given the high dimensionality of the data. We employed MaAsLin 3 v.0.99.4 R package using *normalization = 'NONE'*, *median_comparison_abundance = FALSE*, and *median_comparison_prevalence = FALSE* to identify species-level differential abundance based on absolute abundances normalized using a spike-in bacteria count,[78] as follows:

$$\text{Absolute abundance of a microbe} = \left(\frac{\text{read count of a microbe}}{\text{read count of spike-in}}\right) * \text{spike-in cell count}$$

The read count of a microbe was based on MetaPhlAn4, and the read count of the spike-in microbe was 1.4×10^5 cells of *Vibrio campbellii*. In the MaAsLin model, we also considered 'trial_arm', 'sex', 'ESBL-E', 'born_hospital', and 'antibiotics' as fixed effects.

β-diversity, ordination, and clustering analyses

β-diversity analysis was executed using the *"vegdist"* function from the *vegan* v.2.5.7 R package, applying Bray-Curtis or Jaccard dissimilarity indices, followed by principal coordinate analysis (PCoA) for ordination. To ascertain the statistical significance of composition differences, we conducted a permutational multivariate analysis of variance (PERMANOVA) using the *"adonis"* function from the *vegan* package, with 999 permutations. The Redundancy Discriminant Analysis (RDA) was performed using *vegan* v.2.5.7 R package. The microbiota clusters were generated using an unsupervised non-linear k-means partitioning algorithm (k = 2, *kmean*s function in base R) based on a single variable - *Bifidobacterium* genus relative abundance. In one dimension, k-means estimates two centroids and assigns samples to the nearest centroid, yielding a threshold (midpoint between centroids) that minimizes within-cluster variance. This clustering was used to reflect the primary intervention-associated shift observed at 6 weeks. Ordination was performed using non-metric multidimensional scaling (NMDS) based on Bray-Curtis dissimilarity using *vegan* v.2.5.7 R package. The significance and effect size of each covariate were evaluated using the *'envfit'* function based on 999 permutations from the *vegan* v.2.5.7 R package.

Other statistical analyses and data visualization

All statistical analyses were conducted using R software v.4.4.1. Graphical illustrations were mostly created with the *ggplot2* v.3.3.6 R package. The heatmaps were visualized using *ComplexHeatmap* v.2.20.0 package.[66] Random forest was performed using 10-fold cross-validation, 500 trees, and 1000 permutations using *randomForest* v.4.7.1.2 and *caret* v.6.0.94R packages to identify important predictors of ESBL-E carriage. We employed a generalized linear model with a binomial family using the *glm* function in R to evaluate the impact of various factors on the carriage of ESBL-E. Additionally, we fitted negative binomial models using the *MASS* v.7.3.65 R package. For presence/absence analysis, detection was defined as abundance >0. Absolute counts were converted to per-sample relative abundances by dividing by the column sum. We report detection odds ratios (ORs) with a Haldane–Anscombe 0.5 continuity correction and two-sided Fisher's exact *p*-values. Multiple testing was controlled by Benjamini–Hochberg FDR ($q \leq 0.05$ considered significant).

We employed Spearman's correlation analysis to investigate the relationships between microbiota, resistome, and mobilome diversities using *Hmisc* v.5.1.3 R package, and between the relative abundance of *B. longum* and the richness of potential pathogens using *ggpubr* v.0.6.1 R package. We performed partial least squares path models using *the plspm v.0.5.1 R package to identify pathways among microbial composition, ARGs, and MGEs, thereby quantifying direct and indirect effects among* these components. Pathway coefficient $\beta > 0.3$ was considered a strong interaction. We used DIABLO (Data Integration Analysis for Biomarker Discovery using Latent Variable Approaches for Omics Studies) from the mixOmics v.6.28.082 R package to identify features that underlie associations among the microbiota, resistome, and metabolome. Subsequently, a biweight midcorrelation analysis was conducted using WGCNA v.1.73[68] R package to assess the strength and robustness of associations between the selected features identified by DIABLO. Statistical assumptions were evaluated using the *AssumpSure* v1.0.0[69] R package to ensure transparency in test selection.

Statistical analysis of metabolomic data

Dry-weight normalized untargeted metabolomic data were uploaded to MetaboAnalyst 6.0[62] and filtered using a low-variance filter based on a standard deviation threshold of 40%. The resulting data were median-log10-transformed and Pareto-scaled. Significant features between groups were determined by a Mann-Whitney U test ($p < 0.05$) with unequal group variances, using FDR correction, and visualized as volcano plots with a fold-change cutoff of 2.0 and a *p*-value threshold of 0.05. PCA was performed using the

CellPress
OPEN ACCESS

Euclidean distance matrix, and PERMANOVA was used for statistical testing. For targeted metabolomics, the relative abundance of metabolites was visualized using the R package *ggplot2*, and correlation heatmaps were generated using Spearman correlation with a *p*-value threshold of 0.05.

Computational resources

Bioinformatic analysis of metagenomic data was performed on the Norwegian academic high-performance computing and storage services maintained by Sigma2 Norwegian Research Infrastructure Service (http://www.sigma2.no/, project number: NN8021K).

Cell Reports Medicine, Volume *7*

Supplemental information

Metabolic reprogramming of the infant gut by bifidobacteria-based probiotics drives exclusion of antibiotic-resistant pathobionts

Ahmed Bargheet, Gaute Hovde Bø, Marit Andrea Klokkhammer Hetland, Museveni Justine, Sabrina John Moyo, Iren Høyland Löhr, Bjørn Blomberg, Nina Langeland, Claus Klingenberg, and Veronika Kuchařová Pettersen

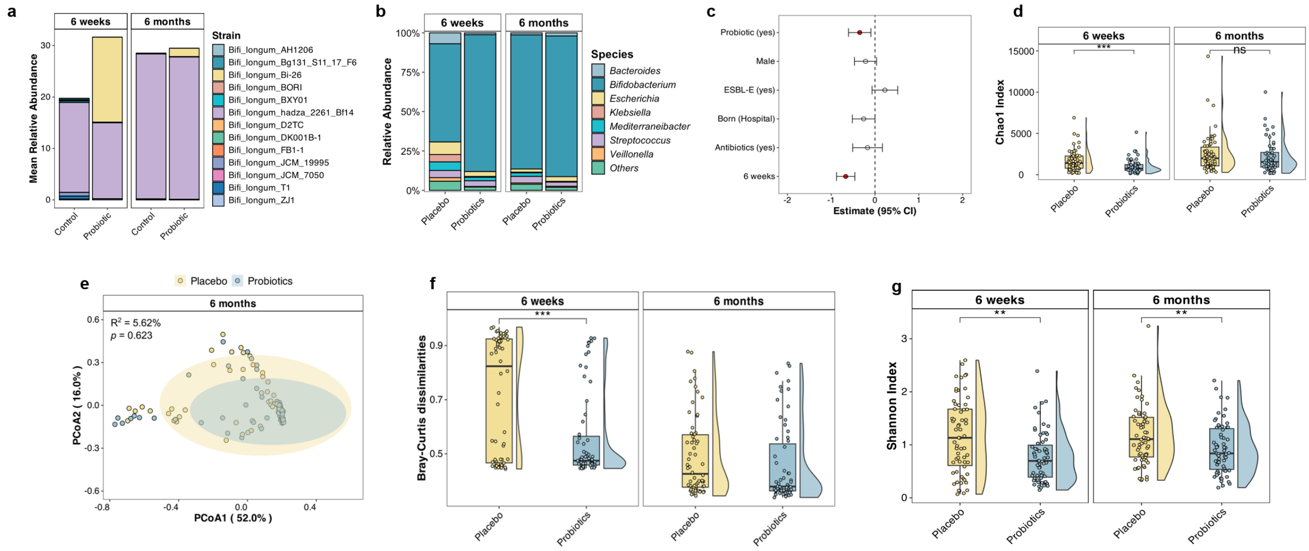

Figure S1. **Analyses of *Bifidobacterium longum* sub-species diversity and microbiota α- and β-diversity.** Related to Figure 1 (a) Relative abundances of *B. longum* strains identified by StrainGE at the 6-week and 6-month time points. (b) Relative abundance of bacterial genera in the placebo and probiotic groups. (c) Impact of selected variables on infant gut microbiota richness, determined by a negative binomial model. (d) Alpha diversity by Chao1 index (Mann–Whitney U test). (e) Principal Coordinate Analysis (PCoA) of Bray–Curtis dissimilarities at 6 months; each point represents one sample. (f) Bray–Curtis dissimilarity to all other samples within the same group at 6 weeks and 6 months; each point represents a sample's mean Bray–Curtis dissimilarity to all other samples in its group (Mann–Whitney U test). (g) Shannon index at 6 weeks and 6 months (Mann–Whitney U test). For Shannon index, the Wilcoxon effect size for 6 weeks = 0.276, magnitude small; for 6 months, effect size = 0.233, magnitude small. Statistically significant results are based on $p < 0.05$. *** $p < 0.001$, ** $p < 0.01$.

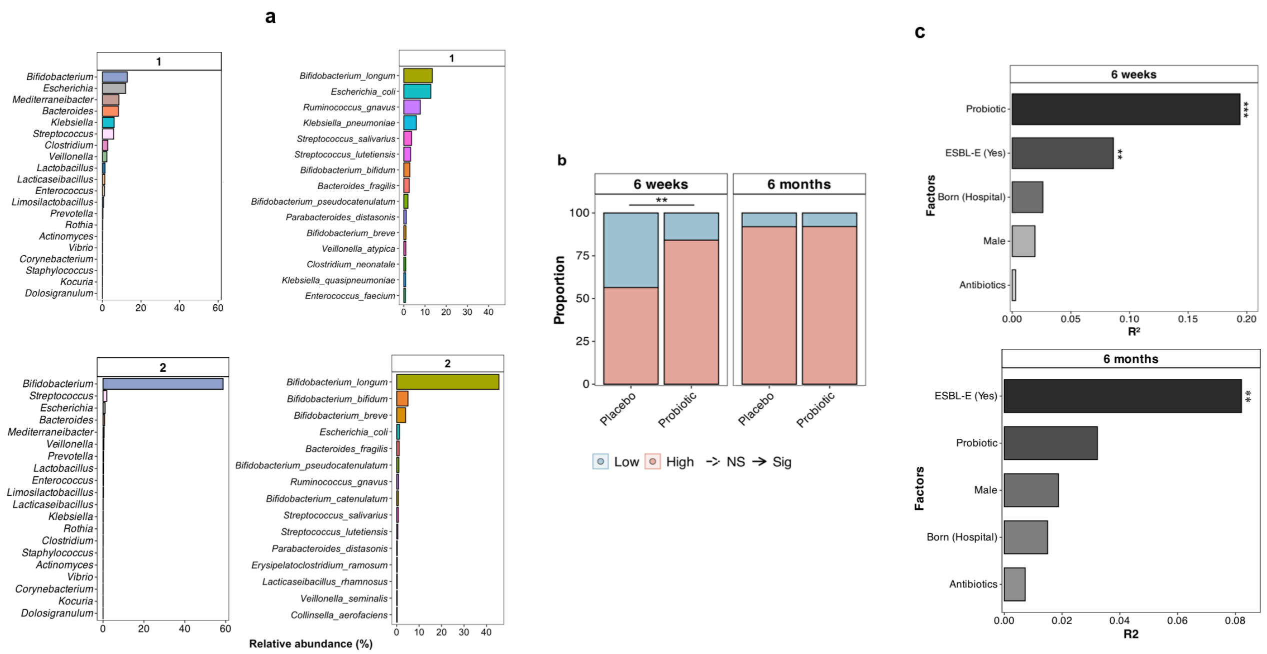

Figure S2. **Unsupervised clustering and Envfit analyses.** Related to Figure 1. a) Microbiota cluster composition identified using k-means clustering, with Cluster 1 ("Low" top row) and Cluster 2 ("High" bottom row) at the genus (left) and species (right) level. (b) Distribution of clusters across groups, with the p-value calculated using Fisher's exact test. (c) Factors associated with microbiota community structure, identified using Envfit analysis, at 6 weeks and 6 months of age. Statistical significance: $p < 0.05$. Symbols: $***$ $p < 0.001$; $**$ $p < 0.01$; $*$ $p < 0.05$.

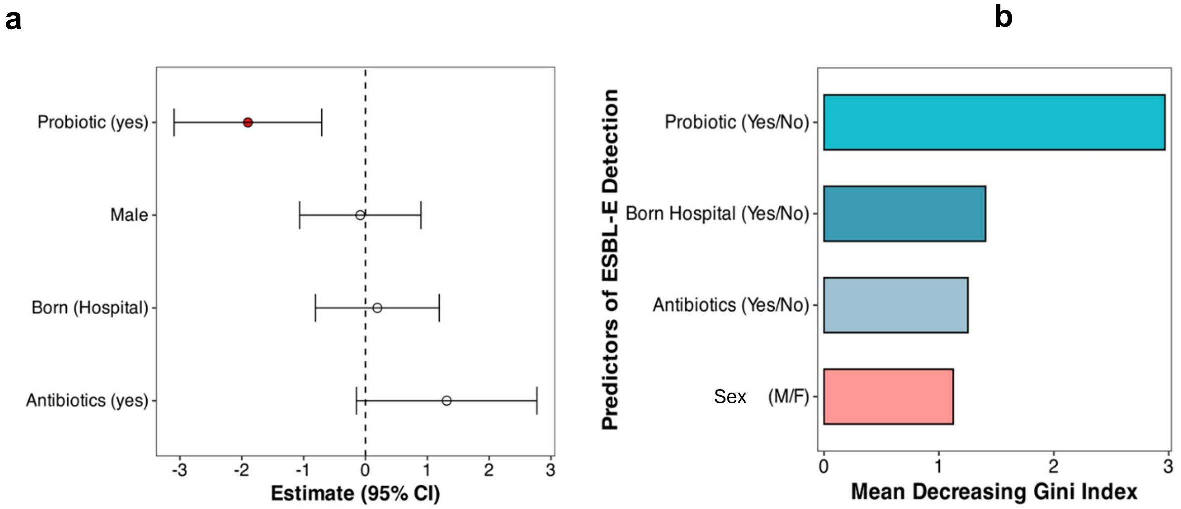

Figure S3. **Impact of probiotics on ESBL-E carriage.** Related to Figure 2. The impact of selected variables on the ESBL-E carriage as determined by generalized linear modelling (a) and random forest (b). The random forest analysis does not indicate which specific binary outcome a variable is associated with, but it identifies which variables are most important for prediction. Statistical significance: $p < 0.05$

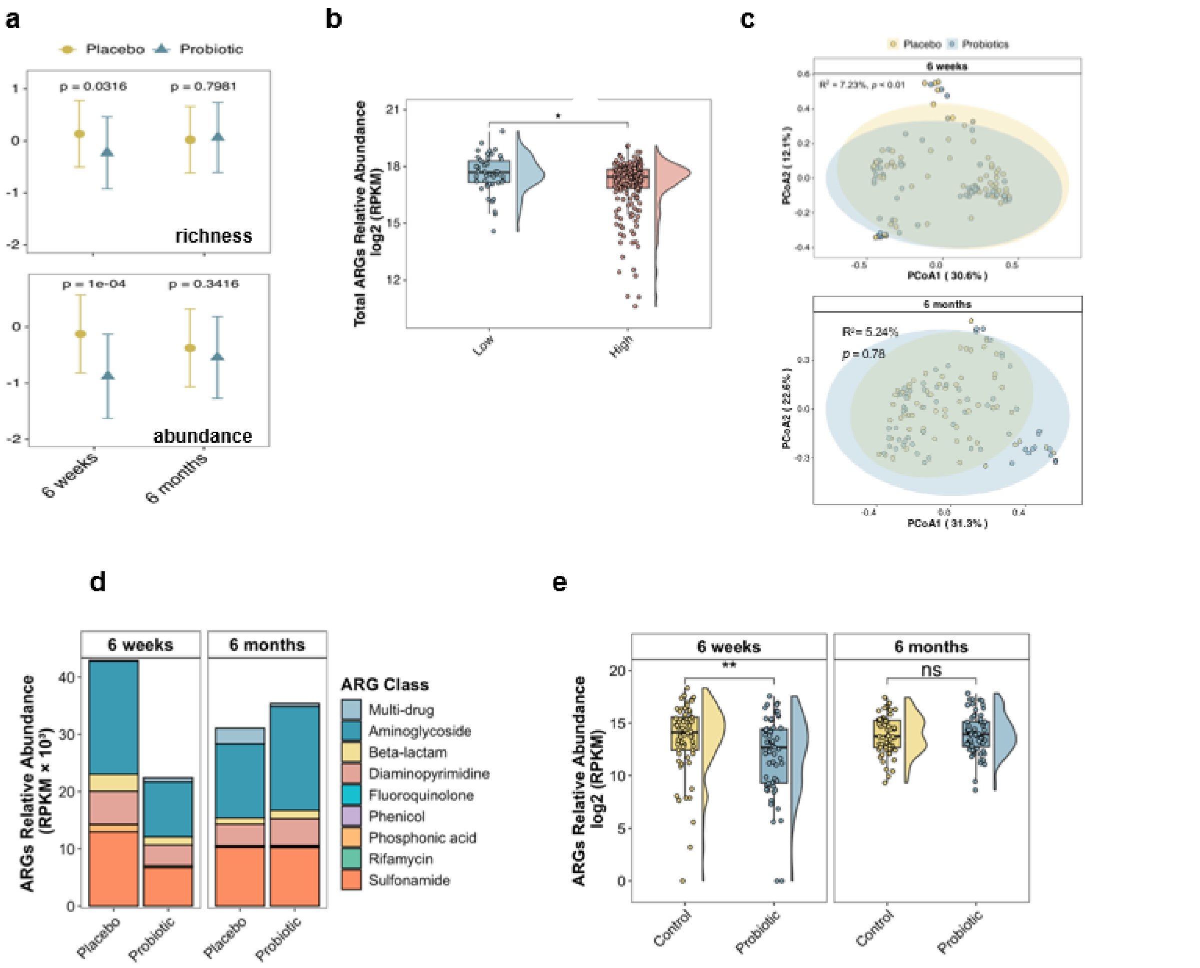

Figure S4. The influence of probiotics on the resistome. Related to Figure 2**.** a) Estimated marginal means of probiotic impact on resistome richness and relative abundance across time points. b) Comparison of antibiotic resistance genes (ARGs) relative abundance between microbiota clusters. Reads per kilobase per million mapped reads (RPKM), with each point representing a sample. p-values were computed using the Mann-Whitney U test. c) Principal Coordinate Analysis (PCoA) illustrating resistome beta diversity differences between the probiotic and placebo groups at 6 weeks and 6 months, visualized through Jaccard dissimilarity comparisons. Panels (d) and (e) reproduce the analyses shown in Figure 2 of the main text but restricted to the 33 clinically relevant ARGs detected in the 32 isolate-metagenome-matched samples. This subset highlights resistome changes attributable specifically to ARGs carried by ESBL-producing isolates recovered from the same individuals. d) Relative abundance of ARGs in RPKM, stratified by ARG classes. The multidrug-resistant class refers to ARGs annotated as conferring resistance to two or more antibiotics. (e) Comparison of ARG abundance between groups, with each point representing a sample. Statistical significance: $p < 0.05$ (∗), ∗∗ $p < 0.01$

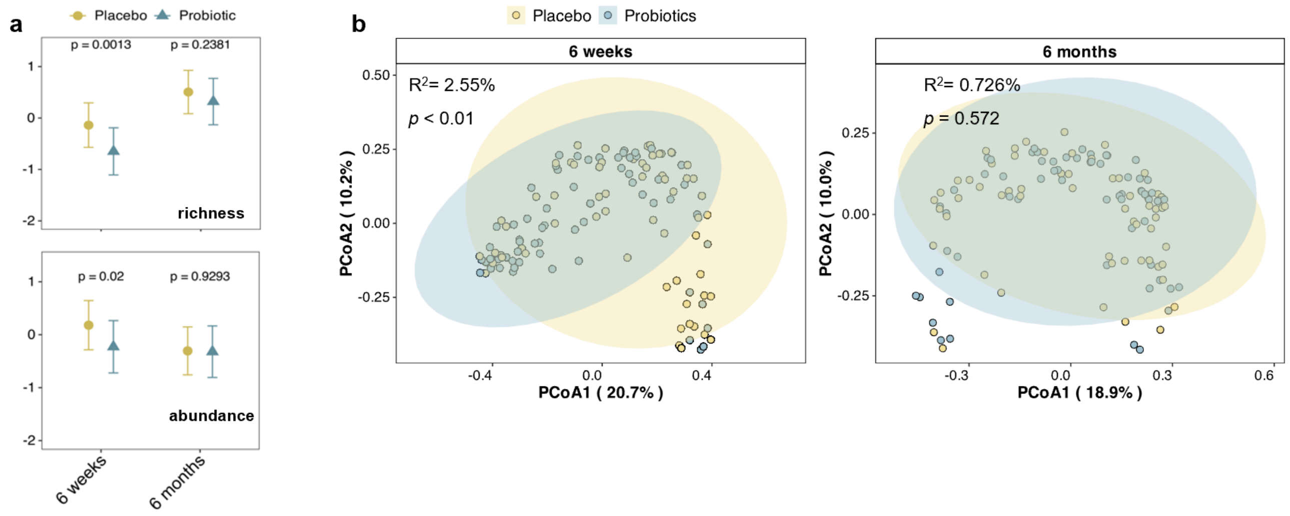

Figure S5. The influence of probiotics on the mobilome. Related to Figure 2. a) Estimated marginal means of probiotic impact on mobilome richness and relative abundance across time points. b) Principal Coordinate Analysis (PCoA) illustrating mobilome beta diversity differences between the probiotic and placebo groups at 6 weeks and 6 months, visualized through Jaccard dissimilarity comparisons. Statistical significance: $p < 0.05$

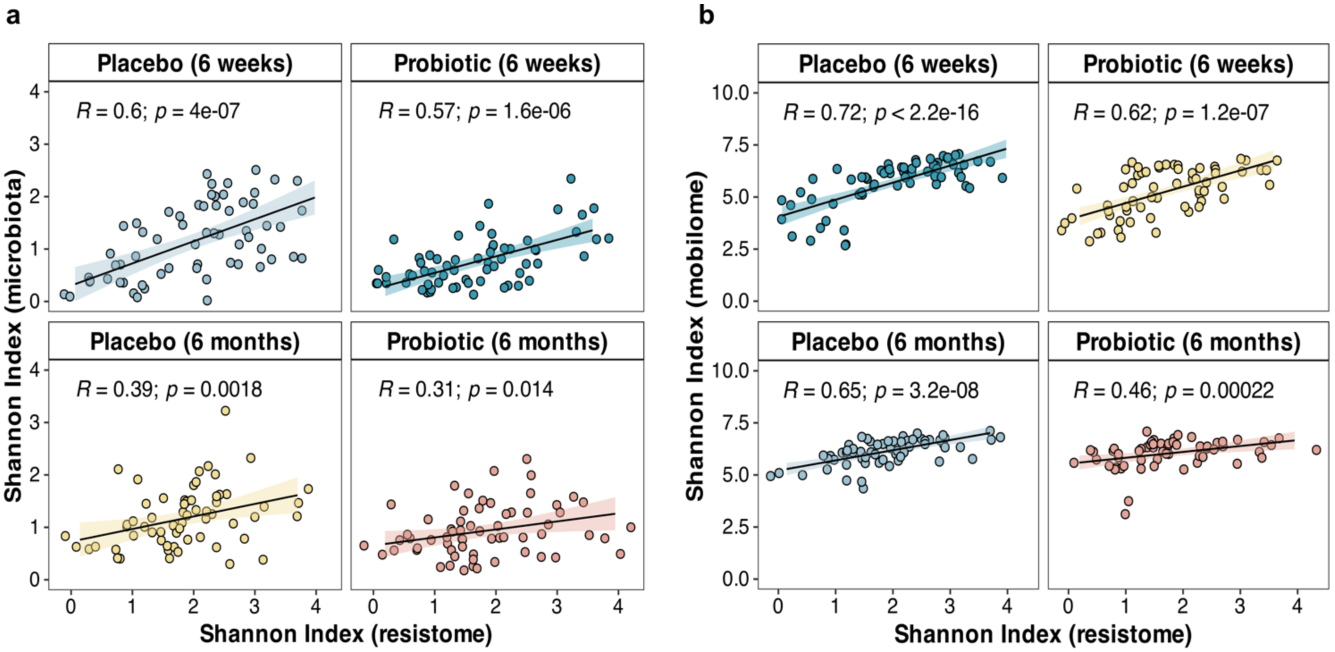

Figure S6. Correlation analysis. Related to Figure **2.** Spearman correlation analysis between resistome diversity and microbiota diversity (a) and between resistome diversity and mobilome diversity (b). The strength of correlation estimated by R values: 0.10-0.30 weak, 0.30-0.50 moderate, 0.5-0.7 strong, 0.7-1 very strong.

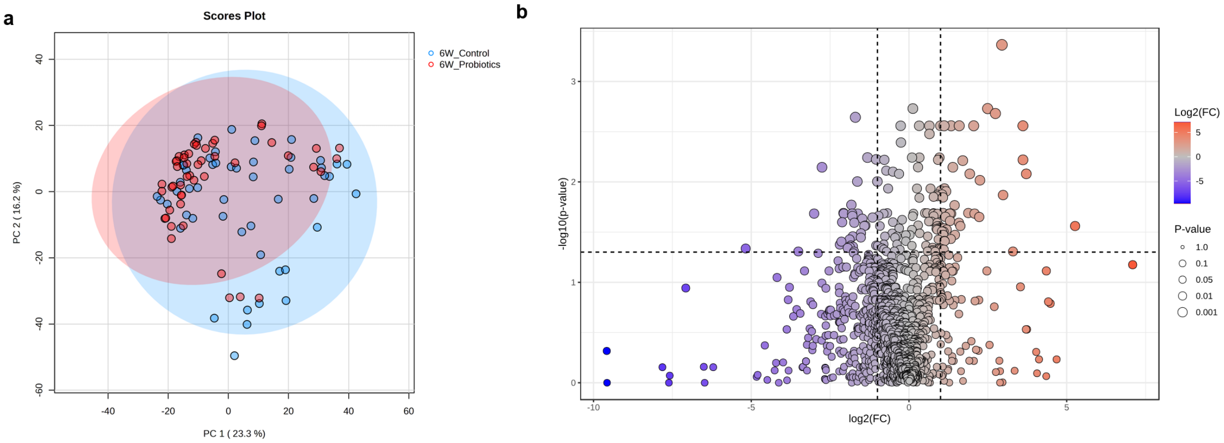

Figure S7. Comparison of the fecal metabolome of placebo and probiotic groups at 6 weeks. Related to Figure 3. a) Principal component analysis (PCA) of 1188 detected metabolic features ($R^2 = 0.07218$, $p = 0.004$) using Euclidean distance matrix. b) Volcano plot describing differences in metabolic features between probiotic and placebo groups. In the probiotic group, 45 features showed significantly increased abundance, whereas 37 showed significantly decreased abundance. Settings used in MetaboAnalyst: *p*-value set to 0.05 (Mann-Whitney U test), accounting for unequal group variance, applying false discovery rate correction, and a fold change cutoff of 2.0. The untargeted metabolomic data were normalized by median, $\log_{10}$ transformed, and Pareto scaled.

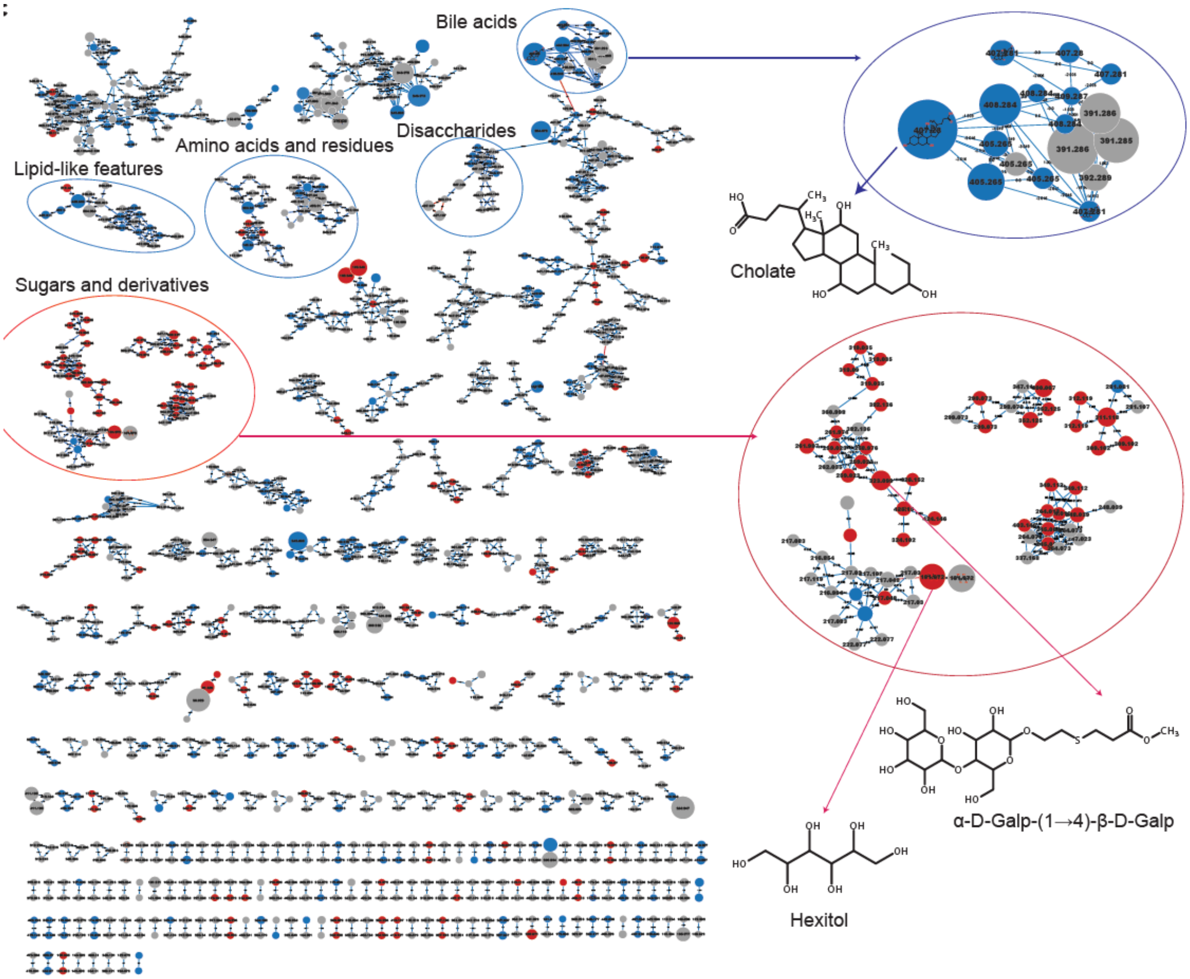

Figure S8 Feature-based molecular networking of untargeted metabolomic data derived from 6 weeks ProRIDE stool samples. Related to Figure 3**.** Clustering was based on cosine and MS1 similarities between features, indicating related fragments and precursor ions, respectively. Node size reflects the sum of the precursor area for each feature. Significantly elevated features in the high Bifidobacterium cluster group are labelled red, while significantly decreased features are labelled blue (Mann-Whitney U test, unequal group variance, false discovery rate, and fold change cutoff 2.0, after median normalization, log10 transformation, and Pareto scaling).

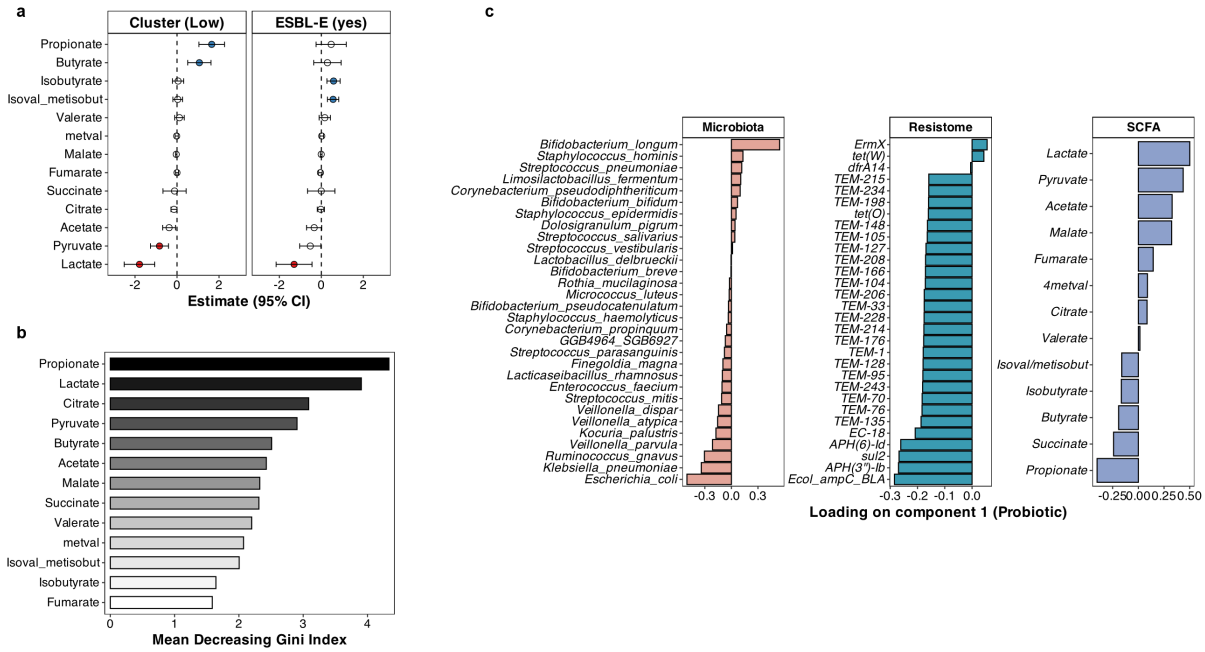

Figure S9 Targeted metabolomics data analysis. Related to Figure 4. **(a)** Differential abundance of the metabolites identified using linear model (Benjamini-Hochberg adjusted p values). (**b**) The importance of SCFAs on microbiota composition as determined by random forest. (**c**) SCFA loadings from DIABLO analysis for the probiotic component.

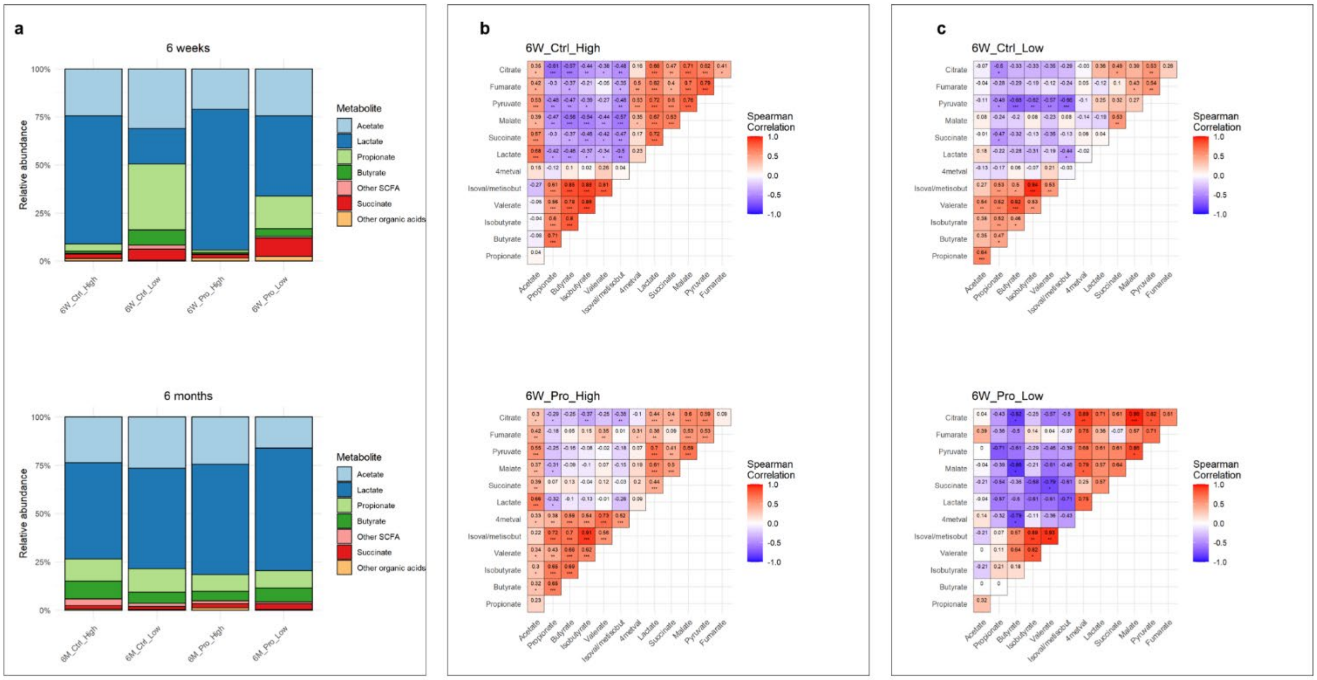

Figure S10. Dynamics of SCFAs and OAs based on *Bifidobacterium* spp. abundance. Related to Figure **4.** a) Relative proportion of SCFAs and organic acids in samples from 6 weeks and 6 months for the probiotic and control group, and with either high or low *Bifidobacterium* spp. abundance. Correlation heatmaps showing Spearman correlations between metabolites for b) “High” and c) “Low” groups at 6 weeks. Asterisks indicate significance levels (*p < 0.05, **p < 0.01, ***p < 0.001).

Table S1. Baseline characteristics of participants. Related to STAR Methods.

Variables	Placebo (N=72)	Probiotics (N=80)
**Mother and delivery**		
Maternal age, median (IQR), years	25 (22.0 - 29.3)	26.5 (22.0 – 31.0)
Maternal HIV infection	2.8 % (2/72)	5.0 % (4/80)
Mode of delivery – n (%)		
• Vaginal	95.8 % (69/72)	97.5 % (78/80)
• Caesarean section	4.2 % (3/72)	2.5 % (2/80)
Birthplace – n (%)		
• Haydom Lutheran Hospital	43.1 % (31/72)	40.0 % (32/80)
• Other health facility	22.2 % (16/72)	25.0 % (20/80)
• At home	34.7% (25/72)	35.0 % (28/80)
**Child**		
Female sex – n (%)	50.0 % (36/72)	48.8 % (39/80)
Birth weight, median (IQR) - kg	3.3 (3.0 – 3.5)	3.3 (3.0 – 3.5)
Birth length, median (IQR) - cm	48.5 (47.0 – 49.0)	48.0 (47.0 – 49.0)
Apgar score at 5 min, median (IQR)*	10 (10-10)	10 (10-10)
Apgar score at 5 min < 9 – n (%)*	0 (0%)	0 (0%)
Apgar score at 5 min		
- 9	3 (4.2 %)	2 (2.5 %)
- 10	27 (37.5 %)	29 (36.3 %)
- Not recorded	42 (58.3 %)	49 (61.3 %)
**Breastfeeding**		
At 6 weeks – no. (%) †	100.0 % (72/72)	100.0 % (80/80)
At 6 months – no. (%) †	94.4 % (68/72)	95.0 % (76/80)
**Caregiver-reported adherence during the 4-week intervention**		
Study drug given daily – no. (%)	84.7 % (61/72)	95.0 % (76/80)
Forgot a dose 1-6 days – no. (%)	13.9 % (10/72)	3.8% (3/80)
Forgot a dose 7-21 days – no. (%)	0.0 % (0/72)	1.3% (1/80)
Forgot a dose ≥ 22 days– no. (%)	1.4% (1/72)	0.0 % (0/80)
**ESBL-E gut colonisation**		
6 weeks	25.0% (18/72)	5.0% (4/80)
6 months	25.0% (18/72)	15.0% (12/80)

Table S1. Baseline characteristics of participants - continuation

Variables	Placebo (N=72)	Probiotics (N=80)
**Death or hospitalisation to 6 m**		
Death* (n, %)	0 (0.0%)	0 (0.0%)
Hospitalisation to 6 m	4 (5.6%)	1 (1.3%)
Δ weight Z birth→6 m, mean (SD)	−0.37 ± 8.10	−0.64 ± 7.60
Δ length Z birth→6 m, mean (SD)	−0.43 ± 1.29	−0.25 ± 1.19
**Caregiver-reported adverse events**		
Gastrointestinal adverse events**	7 (9.7%)	6 (7.5%)
Reported visit to an outpatient clinic (OPC) up to 6 m	23 (31.9%)	22 (27.5%)
OPC ≤6 weeks	8 (11.1%)	8 (10.0%)
OPC 6w–6m	18 (25.0%)	15 (18.8%)
Any antibiotic therapy up to 6 m	23 (31.9%)	27 (33.8%)
Hospitalisation with suspected infection	4 (5.6%)	1 (1.3%)

* No death or blood culture–positive hospitalizations were recorded

** Including diarrhea, constipation, abdominal pain, bloating, and bloody stools

Table S10. Bacterial species with pathogenic potential reported by previous studies. Related to Figure 1.

**Pathobiont**	**Source**
Acinetobacter baumannii	Shao.et,al (2019)[1]; Murray et al., 2022[2]
Campylobacter jejuni	Burnham et al, 2018[3]
Clostridium perfringens	Shao.et,al (2019)
Enterobacter cloacae	Shao.et,al (2019)
Enterococcus faecalis	Shao.et,al (2019); Murray et al., 2022
Enterococcus faecium	Shao.et,al (2019)
Escherichia coli	Murray et al., 2022
Haemophilus influenzae	Murray et al., 2022
Klebsiella oxytoca	Shao.et,al (2019)
Klebsiella pneumoniae	Shao.et,al (2019); Murray et al., 2022
Morganella morganii	Murray et al., 2022
Pseudomonas aeruginosa	Shao.et,al (2019); Murray et al., 2022
Salmonella enterica	Murray et al., 2022
Serratia marcescens	Murray et al., 2022
Staphylococcus aureus	Murray et al., 2022
Staphylococcus epidermidis	Shao.et,al (2019)
Streptococcus parasanguinis	Shao.et,al (2019)
Streptococcus pneumoniae	Murray et al., 2022

REFERENCES

1. Shao, Y., *et al.* Stunted microbiota and opportunistic pathogen colonization in caesarean-section birth. *Nature* **574**, 117–121 (2019).
2. Murray, C.J., *et al.* Global burden of bacterial antimicrobial resistance in 2019: a systematic analysis. *The Lancet* **399**, 629–655 (2022).
3. Burnham, P.M. & Hendrixson, D.R. Campylobacter jejuni: collective components promoting a successful enteric lifestyle. *Nature Reviews Microbiology* **16**, 551–565 (2018).
